# WAVE3-NFκB Interplay Is Essential for the Survival and Invasion of Cancer Cells

**DOI:** 10.1371/journal.pone.0110627

**Published:** 2014-10-16

**Authors:** Gangarao Davuluri, Katarzyna Augoff, William P. Schiemann, Edward F. Plow, Khalid Sossey-Alaoui

**Affiliations:** 1 Department of Molecular Cardiology, Cleveland Clinic Lerner Institute, Cleveland, Ohio, United States of America; 2 Case Comprehensive Cancer Center, Case Western Reserve University, Cleveland, Ohio, United States of America; Michigan State University, United States of America

## Abstract

The WAVE3 cytoskeletal protein promotes cancer invasion and metastasis. We have shown that the WAVE3-mediated activation of cancer cell invasion is due, in part, to its regulation of expression and activity of key metalloproteinases (MMPs), including MMP9, which is centrally involved in invadopodia-mediated degradation of the extracellular matrix (ECM). MMP9 is also a major NFκB target gene, suggesting a potential linkage of WAVE3 to this pathway, which we sought to investigate. Mechanistically, we found that loss of WAVE3 in cancer cells leads to inhibition of NFκB signaling as a result of a decrease in the nuclear translocation of NFκB and therefore loss of activation of NFκB target genes. Conversely, overexpression of WAVE3 was sufficient to enhance NFκB activity. Both pharmacologic and genetic manipulations of NFκB effector molecules show that the biological consequence of loss of WAVE3 function in the NFκB pathway result the inhibition of invadopodia formation and ECM degradation by cancer cells, and these changes are a consequence of decreased MMP9 expression and activity. Loss of WAVE3 also sensitized cancer cells to apoptosis and cell death driven by TNFα, through the inhibition of the AKT pro-survival pathway. Our results identify a novel function of WAVE3 in NFκB signaling, where its activity is essential for the regulation of invadopodia and ECM degradation. Therefore, targeted therapeutic inhibition of WAVE3 will sensitize cancer cells to apoptosis and cell death, and suppress cancer invasion and metastasis.

## Introduction

Metastasis is a complex process requiring cancer cells to escape from their primary site, survive in the blood/lymph system and then to establish a new niche at a distant site [1]. During this process, also referred to as the invasion-metastasis cascade, cancer cells utilize specialized F-actin rich protrusions called invadopodia, to concentrate the enzymatic activity of MMPs to degrade the ECM, thus allowing the cancer cells to invade and migrate through their microenvironment [2], [3]. The WASP/WAVE proteins play central roles in multiple cellular processes, including cell shape, motility, cytokinesis as well as cancer cell invasion [4]–[6]. WAVE3, in particular, has been shown to be essential for the motility and invasion of cancer cells [7]–[9] by contributing to the formation of lamellipodia extensions at the leading edge of invasive cells [8], [10]. The expression of WAVE3 is also strongly enriched in several cancers, including breast cancer (BC) [11]–[14]. In fact, enhanced expression and activity of WAVE3 was shown to contribute the metastasis of triple-negative breast cancers (TNBC), the most aggressive subtype of BC [14]–[16].

Nuclear factor NFκB activation is well known for being implicated in the survival, invasion, and metastasis of various types of cancers [17], [18]. Activation of the NFκB pathway is necessary for diverse physiological and pathological responses ranging from the mounting of a successful immune response and to the survival and proliferation of cancer cells [19]–[21]. The NFκB family of transcriptional factors consists of five members, p50, p52, RelA (p65), RelB and c-Rel, which form homomeric or heteromeric dimers to activate transcription of the target genes [22]. In resting cells, NFκB is maintained in a transcriptionally quiescent state by being sequestered in the cytoplasm in protein complexes with members of the inhibitors of IkappaB (IκB) family, including IκBα, IκBβ, IκBε. In the classical pathway, TNFα can induce IκB kinase (IKK) mediated phosphorylation and proteasomal degradation of IκBα, followed by phosphorylation and nuclear translocation of the p50–p65 heterodimer to activate transcription of NFκB target genes [23]. NFκB has been shown to stimulate the production of MMPs, including MMP1, MMP3, and MMP9 [24]–[26]. Interestingly, we and others have shown that WAVE3 can also regulate the expression and activity of these MMPs, suggesting potential role WAVE3/NFκB interplay in the regulation of MMP9 and invadopodia activity in cancer cells [8], [10].

Here we present evidence that the metastasis promoting activity of WAVE3 is achieved in part through its regulation of NFκB signaling in cancer cells. We show that loss of WAVE3 in the metastatic BC MDA-MB-231 cells results in inhibition of NFκB activity. Conversely, overexpression of WAVE3 enhances NFκB signaling. We show that WAVE3-mediated modulation of NFκB is required for invadopodia formation as well as MMP9 expression and activity that are needed for cancer cells to degrade the ECM. Finally, we show that targeted-inhibition of WAVE3 sensitizes cancer cells to apoptosis and cell death through the inhibition of AKT and caspase survival pathways downstream of NFκB. Accordingly, our data establish a novel function for WAVE3 that is critical for the regulation of NFκB signaling and support the use of WAVE3 inhibitors in combination therapies to specifically target cancer metastasis.

## Experimental Procedures

### Antibodies

Rabbit anti-WAVE3 (1∶1000), rabbit anti-MMP9 (1∶1000), rabbit anti-MMP2 (1∶1000), rabbit anti-p38 (1∶1000), rabbit anti phospho p38 (1∶1000), rabbit phospho ERK (1∶1000), rabbit ERK (1∶1000), rabbit anti phospho AKT (Ser473; 1∶1000), rabbit anti panAKT (1∶1000), rabbit anti Phospho JNK (1∶1000), rabbit anti-JNK (1∶1000), mouse anti-phospho p65 (Ser536) (1∶1000), rabbit anti-Cortatcin are from Cell Signaling Technologies. (Danvers, MA); mouse anti-GFP, mouse anti-WAVE2 (1∶3000), mouse anti-WAVE1 (1∶3000) are from Santa Cruz Biotechnology Inc (Santa Cruz, CA); rabbit anti-NFκB p65 (1∶5000) from Biolegend (San Diego, CA), rabbit anti NAP1 (1∶2000), rabbit anti ABI1 (1∶5000), rabbit anti CYFIP2 (1∶3000), mouse anti-actin (1∶5000), rabbit anti-Myc (1∶1000) are from Sigma-Aldrich (St. Louis, MO); goat horseradish peroxidase-conjugated anti-mouse IgG (1∶5000) and goat horseradish peroxidase-conjugated anti-rabbit IgG (1∶5000) from Calbiochem; and Alexa 488-conjugated anti-rabbit IgG and Alexa 568-conjugated anti-mouse IgG, Alexa 568-conjugated phalloidin are from Invitrogen. Vecta-shield with 4′,6-diamidino-2-phenylindole was from Vector Laboratories (Burlingame, CA). Gel electrophoresis reagents were from Bio-Rad (Hercules, CA).

### siRNA and shRNA gene expression knockdown

We used the On-Targetplus SMARTpool (Thermo Scientific) siRNA L-012141-00-0010, L-1012301-00-0010, CYFIP2HSS120271, ABI1HSS11589 (Invitrogen), and SignalSilence IκBα siRNA (Cell Signaling Technology) for transient knockdown of expression of WAVE2, WAVE3, CYFIP2 and ABI1, respectively, as previously described (14). Stable knockdown of WAVE3 was achieved through transfection of MDA-MB-231 and BT549 BC cells with either the control non-targeting shRNA or the WAVE3 MISSION shRNA clones (Sigma) followed by puromycin selection of positive clones as previously described (12).

### Cell Culture

Human MDA-MB-231 BT549 and MCF7 BC cells were purchased from American Type Culture Collection (ATCC) and were maintained in Dulbecco's modified Eagle's medium (DMEM) supplemented with 10% fetal bovine serum (FBS), 100 units of penicillin/ml, 100 µg of streptomycin/ml. For stimulation with TNFα, the cells were first subjected to serum starvation for overnight in DMEM without fetal bovine serum, and then treated with 50 ng/ml TNFα or the diluent DMSO (basal conditions) as the negative control treatment for 15 min. For protein synthesis inhibition, cell were treated with cycloheximide (CHX; Sigma-Aldrich, St. Louis, MO) at 50 µg/ml and incubated for 24 h. For proteasome inhibition, cells were treated with MG-132 (Sigma-Aldrich, St. Louis, MO) at 20 µM concentration and incubated for 24 h. The Akt-inhibitor MK-2206 (Select Chemicals, Yorktown, TX) was used at the final concentration of 10 µM to treat cells for 16 h while the IKKβ-inhibitor (EMD Millipore, Billerica, MA) was used at the final concentration of 10 µM to treat cells for 24 h.

### Flow cytometry

MDA-MB-231 or BT549 cells, transfected with either the control non-targeting shRNA or the shWAVE3, were treated with TNFα for 3 h and propagated in Dulbecco's modified Eagle's medium (DMEM) culture medium supplemented with 10% FBS and puromycin (5 µg/mL). Subconfluent cell cultures were detached by trypsinization and washed with Hank's Balanced Salt Solution with Ca2+, Mg2+, and 0.1% BSA. Cells were processed for flow cytometry as described by the manufacturer (Roche In-Situ Cell Death Detection Kit, Fluorescein). Propidium Iodide has been used to detect dead cells. All data were acquired in a BD LSRII instrument and analyzed with FlowJo 7.6.3 (Treestar) software. Nonspecific binding of the secondary antibody alone to the cells was subtracted from all flow cytometric data to yield the resultant fluorescence intensity values that are displayed.

### Immunoblot Analysis

Whole cell lysates containing similar amounts of total protein (∼50 µg) were resolved on a 10% sodium dodecyl sulfate-polyacrylamide gel, followed by transfer to nitrocellulose (Bio-Rad, Hercules, CA) or Immobilon-P (Millipore, Billerica, MA) membranes using the Bio-Rad gel and transfer apparatus. Membranes were incubated in 5% whole milk or bovine serum albumin for 1 hour at room temperature, washed with phosphate-buffered saline (PBS), followed by incubation with the primary antibody (as specified) overnight at 4°C. Membranes were then washed and incubated in the appropriate secondary antibody at room temperature for 1 hour, and immunocomplexes were visualized using the Western Lights chemiluminescence detection kit from Perkin-Elmer (Boston, MA). Signals were quantified using the ImageJ software according to the parameters described in ImageJ user guide (http://rsbweb.nih.gov/ij/docs/guide/146.html). Average values from 3 different blots are presented.

### NFκB Luciferase Reporter Assay

Cignal NFκB reporter assay kit from Qiagen Inc (Valencia, CA) was used according to the manufacturer's instructions. Briefly, MDA-MB 231 or BT549 cells were co-transfected with siWAVE3 or siWAVE2 with NFκB reporter, negative and positive controls, along with a Renilla luciferase reporter construct for internal normalization. After 24 h, cells were treated with 50 ng/ml of recombinant human TNFα for 30 min unless otherwise specified. Dual luciferase assays (Promega, Madison, WI) were performed in a 96-well plate using Veritas Microplate Luminometer (Turner Biosystems, Sunnyvale, CA), and promoter activity values are expressed in arbitrary units after normalization to luciferase activity of the Renilla reporter. Experiments were performed in triplicates, and the standard deviations of values are indicated.

### Immunofluorescence Microscopy

Cells were grown on glass coverslips and fixed in 4% paraformaldehyde for 20 min in PBS at room temperature and washed with PBS. The cells were then permeabilized in 0.2% Triton X-100 in PBS for 15 min, washed again with PBS, and incubated in the blocking solution containing 5% bovine serum albumin (Sigma) in PBS for 2 h at room temperature. Primary as well as secondary antibodies were diluted to the recommended concentration in 5% bovine serum albumin in PBS. Cells were incubated with the primary antibody for 1 h, washed with PBS, and then incubated with the secondary antibody for 1 h. Actin filaments (F-actin) were stained with rhodamine-conjugated phalloidin (Molecular Probes, Eugene, OR) in PBS. The coverslips were mounted on object slides using Vectashield mounting medium containing 4′,6-diamidino-2-phenylindole (Vector Laboratories, Burlingame, CA). Fluorescence images were captured using a Nikon TE2000-E inverted microscopy. Signals were quantified using the ImageJ software according to the parameters described in ImageJ user guide (http://rsbweb.nih.gov/ij/docs/guide/146.html). Average values of 5 different images were plotted.

### Gelatin Zymography

Gelatin zymography acrylamide gels (10%) were prepared according to standard procedures [8]. Gelatin was added to the gel solution to a final gelatin concentration of 1 mg/ml. The cell free supernatant was mixed with 2× sample buffer, incubated at room temperature for 10 minutes, and 25 µl of the mixture were loaded onto the gels. After electrophoresis, the gel was incubated in Zymogram renaturing buffer with gentle agitation for 30 minutes at room temperature, followed incubation in the Zymogram developing buffer at 37°C for at least four hours. Gelatinase activity was visualized by staining the gels with Coomassie Brilliant Blue G250 and destained in acetic acid-methanol-dH_2_O (1∶3∶6). Areas of protease activity were photographed with a SLR camera.

### Invadopodia formation Assay

FITC-gelatin degradation assays were performed as per the manufacturer's procedure (Invitrogen). In brief, coverslips (18-mm diameter) were coated with 50 µg/ml poly-L-lysine for 20 min at room temperature, washed with PBS, fixed with 0.5% glutaraldehyde for 15 min and washed with PBS for 3 times. After washing, the coverslips were inverted on a drop of 0.2% FITC conjugated gelatin in PBS containing 2% sucrose, incubated for 10 min at room temperature, washed with PBS for 3 times, quenched with sodium borohydride (5 mg/ml) for 3 min and finally incubated in 2 ml of complete medium for 2 h. Cells (2×10^5^ each well) were plated in FITC gelatin-coated coverslips and incubated at 37°C for 12 h. Invadopodia and ECM degradation were documented using fluorescence confocal microscopy as described [27], [28]. Signal analysis and reconstruction of 3D images were performed using the ImageJ software.

### Statistical analyses

The data are presented as means ± standard deviations of at least three independent experiments. The results were tested for significance using an unpaired Student's *t* test. A *p* value less than 0.05 was considered significant.

## Results

### WAVE3 is required for NFκB activity

**Figure 1:**
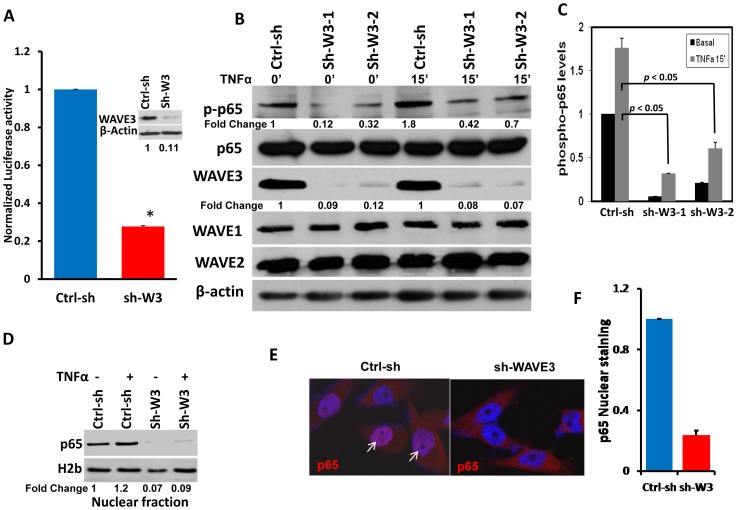
WAVE3 is required for NFκB activation. (A) Luciferase-based NFκB reporter assay in MDA-MB-231 cells with stable transfection of a non-targeting shRNA (Ctrl-sh) or the WAVE3-trageting shRNA (sh-W3). (Inset) Western blot analysis of protein lysates of cells described in (A) with anti-WAVE3 antibody. β-Actin was used as a loading control. (*, p<0.05). (B) Western blot analysis with the indicated antibodies of protein lysates from Ctrl-sh MDA-MB-231 and two different shWAVE3-derived clones (sh-W3-1 and sh-W3-2), before and after TNFα treatment (50 ng/μl for 15 min). The numbers below the p-p65 and WAVE3 panels indicate the fold change of p-p65 and WAVE3 levels, respectively, as compared to the untreated Ctrl-sh cells. (C) Quantification of p-p65 levels in the indicated conditions. (D) Western blot analysis with p65 antibody of the nuclear fraction lysates from the Ctrl-sh and the sh-W3 MDA-MB-231 cells, with or without TNFα treatment. H2b was used as a loading control for the nuclear fraction. The numbers below the H2b panel indicate the fold change p65 levels with respect to the untreated Ctrl-sh cells. (E) Immuno-staining for nuclear translocation (white arrows) of p65 protein (Red) in Ctrl-sh and shWAVE3 MDA-MB-231 cells. Cells nuclei are counter-stained with DAPI (Blue). (F) Quantification of p65 nuclear staining. All data are representative of 3 independent experiments, or are the mean ± SD (n = 3; *, p <0.05; Student's t-test)

To document the interplay between WAVE3 and NFκB signaling, we used a luciferase-based NFκB reporter assay to assess changes in NFκB activity in the presence and absence of WAVE3. As a positive control for NFκB activation, we used TNFα, a known stimulator of NFκB signaling. We found that TNFα treatment of MDA-MB-231 or BT549 cells increased NFκB activity by at least 3-fold in both cell lines as compared to unstimulated cells (Fig. S1 in File S1). However, in the WAVE3-knockdown (sh-W3) cells (Fig. 1A inset), the luciferase activity, and therefore NFκB activity, was reduced by at least 4-fold compared to MDA-MB-231 transfected with control non-targeting shRNA (Ctrl-sh) (Fig. 1A). Thus, WAVE3 was required for NFκB activity. This inhibition of NFκB activity was specific to the loss of WAVE3 since knockdown of WAVE2 expression, another WAVE isoform, had no effect on of NFκB activity (Fig. S2 in File S1).

Phosphorylation of the NFκB p65 subunit at serine 536 and its nuclear translocation are requisite steps for NFκB activation. We found loss of WAVE3 in MDA-MB-231 cells to significantly inhibit (3- to 8-fold decrease) p65 phosphorylation levels (Fig. 1B and 1C). Even after stimulation with TNFα, p65 phosphorylation levels in WAVE3-knockdown cells could not be restored to those levels found in non-targeting shRNA-transfected cells (Fig. 1B and 1C). Similar results were found in BT549 cells (Fig. S3 in File S1). Additionally, nuclear translocation of p65, which is the ultimate step in the activation cascade of NFκB signaling, was also significantly inhibited in WAVE3-knockdown MDA-MB-231 cells, as measured by the levels of p65 protein in the nucleus (10-fold decrease) by both immunoblotting of the nuclear fraction of cell lysates (Fig. 1D) and immunostaining analyses (5-fold decrease) of whole cells (Fig. 1E, 1F and Fig. S4 in File S1). Again, stimulation of WAVE3-knockdown cells with TNFα failed to increase p65 within the nuclear fraction to levels found in the control cells transfected with the control non-targeting shRNA (Fig. S4 in File S1). Together, these data further support the involvement of WAVE3 in the activation of the NFκB signaling pathway.

### WAVE3 overexpression activates NFκB signaling activity

**Figure 2:**
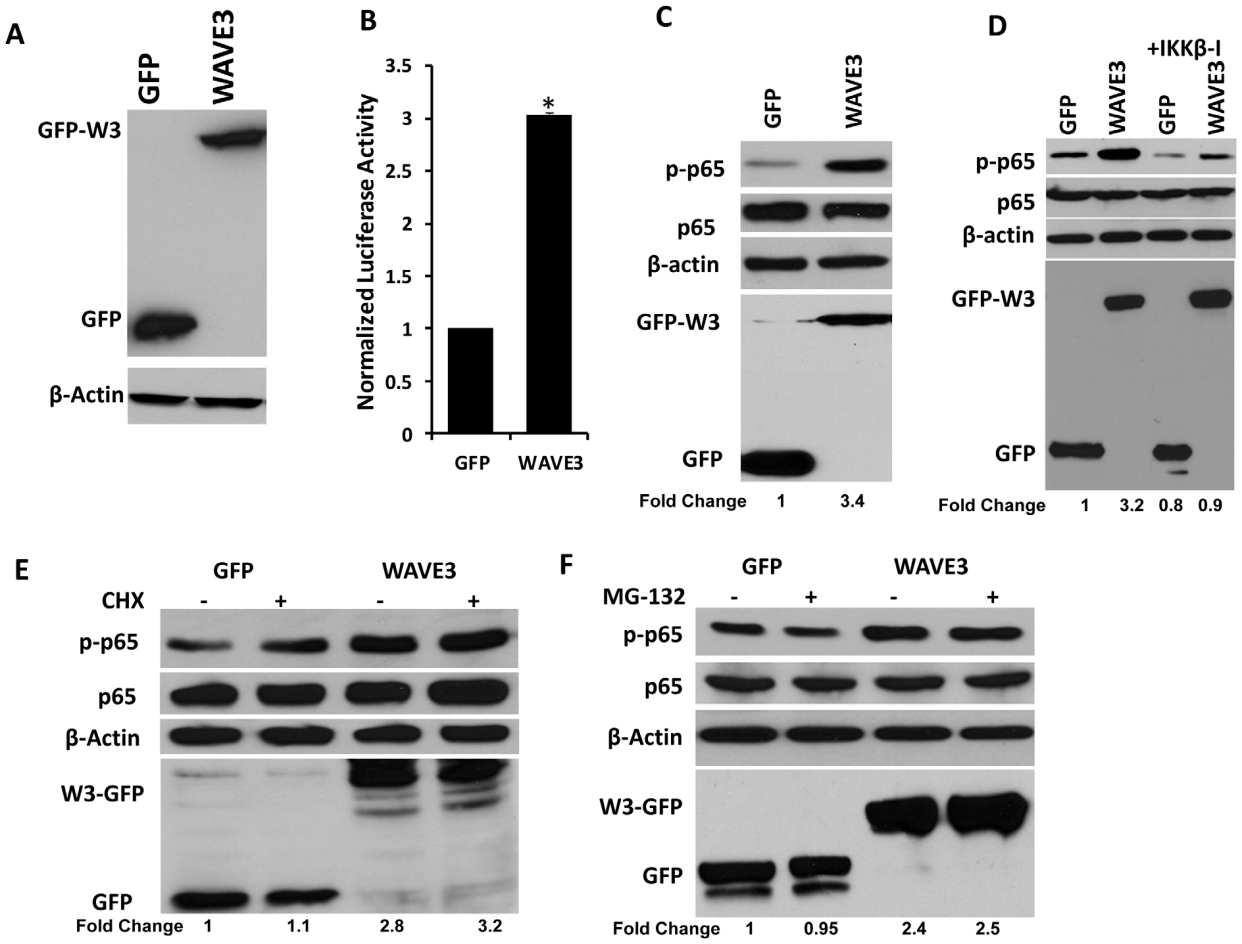
Over-expression of WAVE3 activates NFκB signaling. (A) Western blot analysis of WAVE3-GFP protein levels in GFP and GFP-W3-expressing cells. (B) Luciferase-based NFκB reporter assay in GFP and GFP-WAVE3 expressing cells (*, p<0.05). (C & D) Western blot analysis with the indicated antibodies of cell lysates from the GFP and WAVE3-GFP expressing cells. The numbers below the GFP panel indicate the fold change p-p65 levels with respect to the GFP cells. (E & F) Western blot analysis with the indicated antibodies of cell lysates from the GFP and WAVE3-GFP expressing cells after treatment with cyclohexamide (CHX, E) and the proteasome inhibitor MG132 (F). The numbers below the GFP panel indicate the fold change p-p65 levels with respect to the GFP cells. β-Actin was used a loading control. All data are representative of 3 independent experiments, or are the mean ± SD (n = 3; *, p <0.05; Student's t-test)

To better understand how WAVE3 modulates NFκB signaling, we applied a combination of genetic and pharmacologic means to manipulate effector molecules in the NFκB pathway. First, we examined whether overexpression of exogenous WAVE3 can affect NFκB activity. To do so, we overexpressed either GFP alone or GFP-WAVE3 fusion protein in MDA-MB-231 cells and assessed for NFκB activity. Using the luciferase-based NFκB reporter assay described in Figure 1A, we found overexpression of WAVE3 (Fig. 2A) stimulated NFκB activity by 3-fold increase (Fig. 2B). Consistent with this observation, WAVE3-overexpresion also stimulated (>3-fold increase) p65 phosphorylation without affecting total p65 expression levels (Fig. 2C). Conversely, treatment with a specific inhibitor of IKKβ (IKKβ-I), the upstream activator of NFκB signaling, blunted the TNFα-mediated stimulation of phospho-p65 in the WAVE3-overexpressing MDA-MB-231 cells (0.9-fold change in the TNFα and IKKβ-I-treated cells vs. 3.2-fold change in TNFα only treated cells (Fig. 2D)). Thus, these data further support the role of WAVE3 in the regulation of NFκB signaling. Next, we monitored p65 protein phosphorylation and turnover in the GFP and WAVE3-overexpressing MDA-MB-231 cells after treatment with cyclohexamide (CHX), a potent inhibitor of *de novo* protein synthesis. While phosphorylation levels of p65 were increased (∼3- to 4-fold increase) in the WAVE3-overexpressing cells, treatment with CHX did not affect the expression levels of total p65 (Fig. 2E). Likewise, treatment with the MG-132, a compound that inhibits the proteasome-mediated protein degradation, did not affect the WAVE3-mediated increase of phosphorylated p65 in the WAVE3 overexpressing cells (Fig. 2F) while the levels of total p65 remained unchanged (Fig. 2F). Thus, these data demonstrate that the WAVE3-mediated modulation of NFκB signaling does not require the involvement of WAVE3 in protein neo-synthesis (the CHX data, Fig. 2E) or t proteasome-mediated protein degradation (the MG-132 data, Fig. 2F).

### WAVE3-mediated modulation of NFκB signaling does not require other members of the WAVE regulatory complex

**Figure 3:**
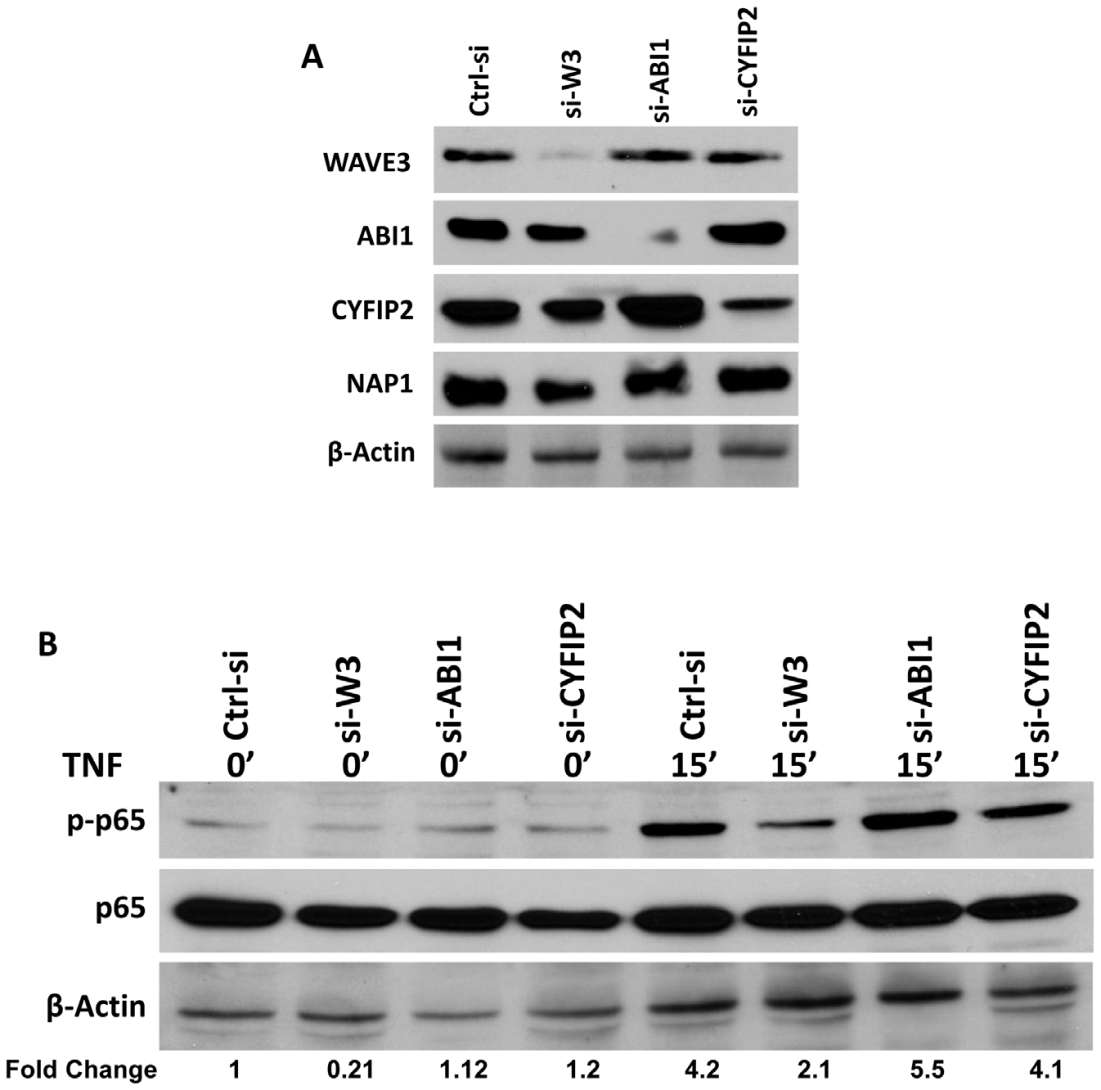
The WAVE3-mediated modulation of NFκB signaling does not require other members of the WAVE complex. (A) Western blot analysis with the indicated antibodies of MDA-MB-231 cells transiently transfected with a non-targeting siRNA (Ctrl-si) or siRNA targeting either WAVE3 (si-W3), ABI1 si-ABI1), or CYFIP2 (si-CYFIP2). (B) Western blot analysis with p-p65 and total p65 antibodies from the cell lysates described in (A). The numbers below the β-Actin panel indicate the fold change p-p65 levels with respect to the untreated Ctrl-si cells. In both (A) and (B), β-Actin was used as a loading control.

WAVE3, like the other members of the WASP/WAVE family, is known to function as an activator of the Arp2/3 complex to promote actin polymerization and cytoskeleton remodeling. For WAVE proteins to execute this cytoskeletal reorganization function, it must be incorporated into a pentameric protein complex, also referred to as the WAVE regulatory complex (WRC) that, in addition to WAVE proteins, also contains NAP1, ABI1/2, CYFIP2 and HSPC300 [29]. Accordingly, we addressed the question as to whether the WAVE3-mediated modulation of NFκB signaling requires the involvement of the other members of the WRC. First, we showed that while siRNA-mediated knockdown of WAVE3 resulted in a significant inhibition (>8-fold decrease) of WAVE3 expression, it did not affect the expression levels of the other four members of the WRC (Fig. 3A). Conversely, siRNAs directed against either ABI1 or CYFIP2, which resulted in selective loss of expression of their respective targets, did not affect the expression levels of the other members of the WRC (Fig. 3A). Functionally, and as expected, WAVE3-knockdown inhibited NFκB signaling (5-fold decrease), as measured by the loss phosphorylation of p65 (Fig. 3B). Nevertheless, decreased expression of any of the other members of the WRC (ABI1 and CYFIP2) did not affect the phosphorylation levels of p65 (Fig. 3B). Furthermore, while stimulation with TNF increased p65 phosphorylation levels in both control and knockdown cells, phosphorylation levels in the WAVE3-knockdown cells were more than two-fold less than in the control siRNA cells or ABI or CYFIP2 siRNA cells (Fig. 3B). Together, these results clearly suggest that the WAVE3-mediated modulation of NFκB signaling does not require the participation of the members of the WRC, and therefore, represents an activity of WAVE3 independent of its traditional actin polymerization property within the WRC.

### Expression and activity of MMP9, a major NFκB target gene, are inhibited by loss of WAVE3

**Figure 4:**
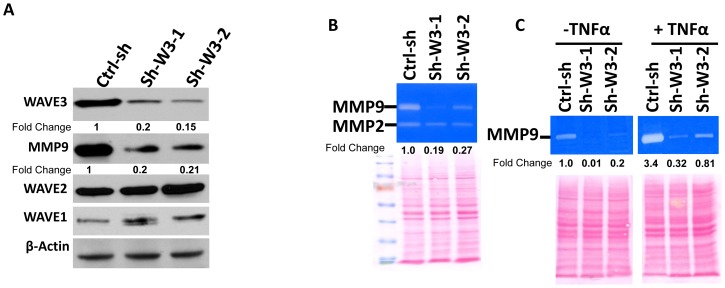
Loss of WAVE3 inhibits the NFκB-mediated stimulation of MMP9 expression and activity. (A) Western blot analysis with the indicated antibodies of cell lysates of MDA-MB-231 cells transfected with non-targeting shRNA (Ctrl-sh), and two different sh-WAVE3 clones (sh-W3-1 and sh-W3-2). The numbers below the WAVE3 and MMP9 panels indicate the fold change WAVE3 and MMP9 levels with respect to the untreated Ctrl-sh cells. β-Actin was used as a loading control. (B) Gelatin zymography of activated MMP9 and MMP2 in conditioned media of the Ctrl-sh two sh-W3 clones. C) Gelatin zymography of activated MMP9 before and after treatment with TNFα in the conditioned media of the Ctrl-sh and two sh-W3 clones. In both (B) and (C), the Red-Ponceau-stained gels are shown as loading controls. The numbers below the zymography panels indicate the fold change of MMP9 levels with respect to the untreated Ctrl-sh cells.

We have previously shown that stable knockdown of WAVE3 results in the loss of expression and activity of several MMPs, including MMP1, 3 and 9 [8]. Among these MMPs, MMP9 is a major target of the NFκB pathway, raising the possibility that WAVE3 might be involved in NFκB-mediated regulation of MMP9. First, we used Western blotting to establish that MMP9 expression was inhibited as a result of stable knockdown of WAVE3; the decrease in MMP9 protein was ∼5-fold in MDA-MB-231 (Fig. 4A) and ∼4-fold in BT549 cells (Fig. S5 in File S1). Of note, expression levels of the WAVE1 and WAVE2 were not affected by loss of WAVE3, and therefore have no bearing on the changes of MMP9 levels caused by WAVE3-knockdown (Fig. 4A). Next, we used the gelatinase zymography assay to show MMP9 activity was also inhibited (5-fold decrease) in the WAVE3-knockdown cells (Fig. 4B). Additionally, stimulation with TNFα, which is a major activator of the NFκB signaling pathway, enhanced (∼3.5-fold increase) MMP9 activity in the control cells (Ctrl-sh). However, in WAVE3-knockdown cells, stimulation with TNFα was unable to activate MMP9 to the levels seen in the control cells (Fig. 4C). Thus, these results suggest that the WAVE3-mediated regulation of MMP9 may be exerted through modulation of NFκB signaling. Therefore, expression and activity levels of MMP9 may be used as readout for WAVE3-mediated modulation of NFκB activation levels.

### WAVE3 is required for invadopodia formation and ECM degradation through NFκB signaling

**Figure 5:**
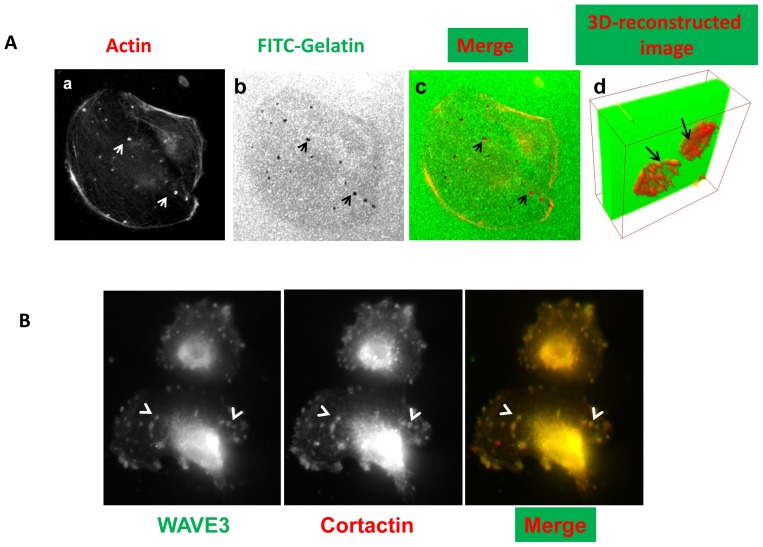
WAVE3 is involved in invadopodia formation and ECM degradation. (A) Confocal microscopic micrographs of MDA-MB-231 cells grown on FITC-labeled gelatin and stained for (a) F-actin filaments (red). The white arrow-heads point to invadopodia structures (white spots). (b) Areas of ECM degradation (black arrow-head) are shown as black spots. The invadopodia structures coincide with the areas of ECM degradation in the merged image (c). (d) In the 3-dimensional reconstructed image the black arrows point to invadopodia (red) infiltrating the gelatin bed (green). (B) Confocal microscopic micrographs of MDA-MB-231 cells grown on collagen-coated coverslips, treated with TNFα (50 ng/μl) for 15 min, and stained for WAVE3 (left panel) and Cortactin (middle panel). The arrow-heads point to areas with invadopodia where both Cortactin and WAVE3 colocalize in the merged image in the right panel (yellow spots).

The biological significance of loss of WAVE3 and its effect on the NFκB signaling were investigated through the ability of cancer cells to form invadopodia and degrade the ECM, both of which require MMP9 activation driven by NFκB signaling. MDA-MB-231 cells, like most highly invasive cancer cell lines, form invadopodia *in vitro* when seeded onto components of the extracellular matrix. To monitor the MMP9 activity within invadopodia, MDA-MB-231 cells were coated onto fluorescent gelatin-coated coverslips. After staining for F-actin, invadopodia were observed as dot-like clusters of F-actin on the ventral surface of the cells that is in direct contact with the gelatin substratum (Fig. 5A panel a). These invadopodia structures overlap with sites of degradation of the gelatin matrix (Fig. 5A, panels b and c) and can be visualized in 3-dimensional (3-D) reconstructed images as invaginations in the gelatin bed (Fig. 5A panel d). Cortactin is a well-known marker for invadopodia (Fig. S6 in File S1). We found that TNFα treatment of MDA-MB-231 resulted in co-localization of WAVE3 with Cortactin at invadopodia structures (Fig. 5B), consistent with the involvement of WAVE3 in invadopodia formation.

**Figure 6:**
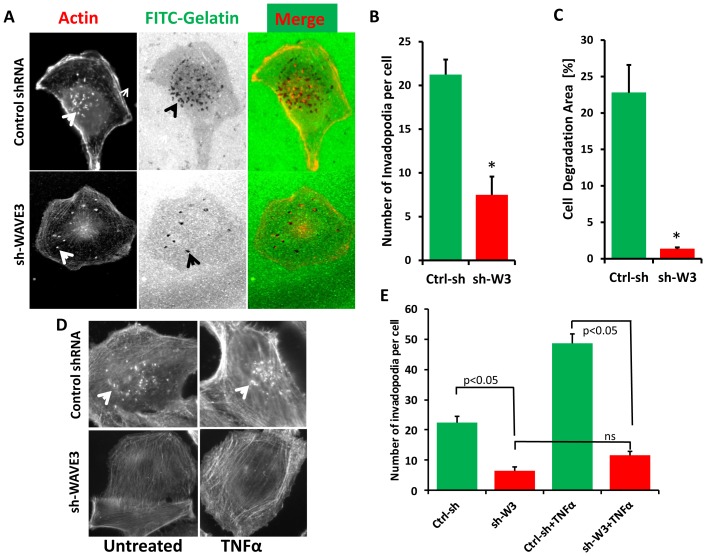
WAVE3 is required for invadopodia formation and ECM degradation through NFκB signaling. (A) Confocal microscopy micrographs of control shRNA- or sh-WAVE3-expressing MDA-MB-231 cells grown on FITC-labeled gelatin and stained for F-actin filaments (left panels). The white arrow-heads point to invadopodia structures (white spots). Areas of ECM degradation (black arrow-heads) are shown as black spots (middle panels). The invadopodia structures coincide with the areas of ECM degradation in the merged image (right panels). (B) Quantification of number of invadopodia per cell in the control shRNA and shWAVE3 cells. (C) Quantification of area of gelatin degradation per cell in the shRNA and shWAVE3 cells. (D) Confocal microscopy micrographs of control shRNA- or sh-WAVE3-expressing MDA-MB-231 cells grown on FITC-labeled gelatin and stained for F-actin filaments with or without treatment with TNFα (50 ng/μl for 15 min). (E) Quantification of number of invadopodia formed in the shRNA and shWAVE3 cells with or without treatment with TNFα. All data are representative of 3 independent experiments, or are the mean (±SE; n = 3; *, **, p <0.05; Student's t-test).

We used this gelatin-based invadopodia assay to assess the effect of WAVE3 on the formation of invadopodia and subsequently the degradation of the ECM by MDA-MB-231 cells. We found a significant reduction of both the number of invadopodia (Fig. 6A, left panel and Fig. 6B), as well as the total area of invadopodia-mediated degradation of gelatin in WAVE3-knockdown cells compared to the non-targeting shRNA control (Ctrl-sh) MDA-MB-231 cells (Figure 6A, middle panel and Fig. 6C). There was more than 3-fold reduction (p<0.05) in the number of invadopodia (Fig. 6B) and more than 10-fold reduction (p<0.05) in the area of ECM degradation in the WAVE3-knockdown cells compared to the control cells (Fig. 6C). While Our data show that loss of WAVE3 inhibits both invadopodia formation and degradation of ECM, this phenomenon seems to affect the majority of the cells observed under the microscope, and careful analysis of our data did not find a decrease in the number of cells that form invadopodia and degrade the ECM, rather, loss of WAVE3 seems to have a global effect. Treatment with TNFα, which resulted in a significant increase in the number of invadopodia in the control cells (p<0.01), was unable to rescue the inhibition of invadopodia caused by loss of WAVE3 (Fig. 6D & 6E). Conversely, over-expression of WAVE3 in the non-invasive MCF7 BC cells (Fig S7A in File S1), which we had previously shown to stimulate their invasiveness [30], resulted in a clear increase in both invadopodia formation and gelatin degradation by TNFα-stimulated cells (Fig. S7B in File S1).

These results demonstrate a positive correlation between WAVE3 expression levels in BC cells and their potential for invadopodia formation and ECM degradation. Collectively, our data also demonstrate the role of WAVE3 in the modulation of the NFκB signaling and its downstream biological effects on MMP9- and invadopodia-mediated regulation of the ECM degradation that is required for cancer cell motility and invasion.

### The WAVE3:NFκB interplay also involves Akt signaling to regulate invadopodia and ECM degradation in cancer cells

**Figure 7:**
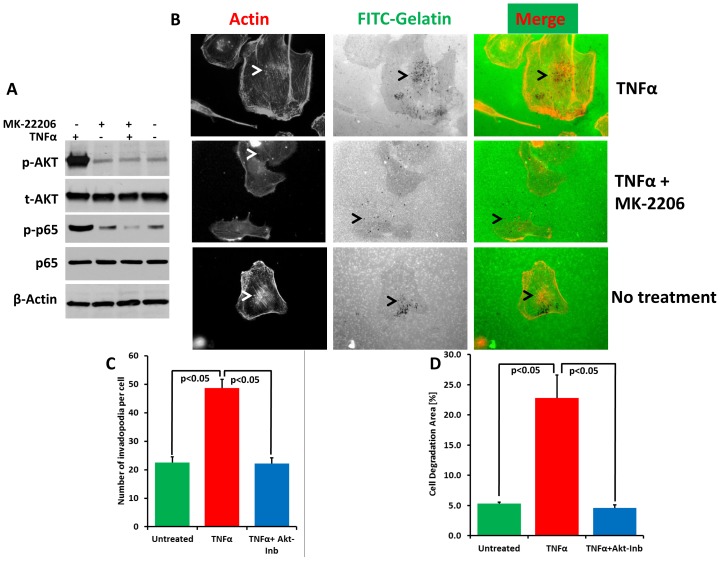
The WAVE3:NFκB interplay involves Akt signaling to regulate invadopodia and ECM degradation in cancer cells. (A) Western blot analysis with the indicated antibodies of cell lysates of untreated MDA-MB-231 cells or treated with TNFα, MK-2206 or both. β-Actin was used a loading control. (B) Confocal microscopy micrographs of MDA-MB-231 cells grown on FITC-labeled gelatin, treated as indicated and stained for F-actin filaments (left panels). The white arrow-heads point to invadopodia structures (white spots). Areas of ECM degradation (black arrow-heads) are shown as black spots (middle panels). The invadopodia structures coincide with the areas of ECM degradation in the merged image (right panels). (C) Quantification of number of invadopodia per cell in the control and treated cells. (D) Quantification of area of gelatin degradation per cell in the control and treated cells. All data are representative of 3 independent experiments, or are the means ± SD.

A recent study by Yamaguchi and colleagues [31] showed that the activity of phosphoinositide 3-kinase (PI3K) is required for invadopodia formation in cancer cells. Specifically, they showed that inhibition of Akt signaling, a downstream effector of PI3K, suppressed invadopodia formation induced by constitutively active mutant PI3K [31]. Interestingly, our previously published work [7] established that a direct interaction between WAVE3 and the p85, the regulatory subunit of PI3K, is required for the PI3K modulation of the WAVE3-mediated regulation of lamellipodia in cancer cells [7]. We, therefore, sought to determine whether the PI3K signaling, and specifically its downstream effector Akt, is also involved in the WAVE3-mediated regulation of invadopodia formation and ECM degradation. Overexpression of WAVE3 in MCF7 cells also resulted in an increase of phosphorylated levels of Akt (Fig. S7C in File S1). Further studies were performed to explore the relationship between WAVE3 and Akt We used the small molecule inhibitor MK-2206, an allosteric kinase inhibitor of Akt [32]–[35] to inhibit Akt activity. Treatment of MDA-MB-231 cells with MK-2206 resulted in a significant reduction in both phospho-Akt levels as well as phospho-p65 induced by TNFα, without affecting total Akt or total p65 levels (Fig. 7A). Thus, while Akt signaling is activated by NFκB (TNFα treatment), NFκB signaling is in turn negatively affected by Akt inhibition (MK-2206 treatment). Inhibition of Akt signaling resulted in a significant inhibition of both invadopodia formation (Fig. 7B and 7C) and gelatin degradation induced by TNFα (Fig. 7B and 7D). These results confirm the findings of Yamaguchi et al. [31], and, more importantly show that the Akt signaling is also involved in the WAVE3-mediated regulation of invadopodia formation and ECM degradation induced by NFκB signaling.

### Down regulation of WAVE3 sensitizes MDA-MB-231 cells to apoptosis and cell death

**Figure 8:**
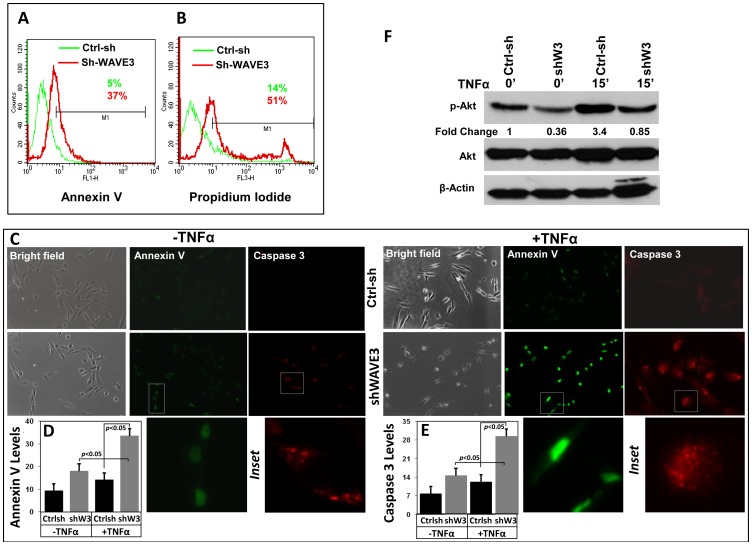
Down-regulation of WAVE3 sensitizes MDA-MB-231 cells to TNFα induced apoptosis through Akt signaling. Representative histograms using flow cytometry of control shRNA (ctrl-sh, green)- or sh-WAVE3-expressing (red) MDA-MB-231 cells after TNFα treatment stained by Annexin V for apoptosis (A) and by Propidium Iodide for cell death (B). (C) Representative confocal images of Ctrl-sh and sh-W3 MDA-MB-231 cells stained Annexin V (Green) and cleaved caspase3 (Red) before and after TNFα treatment (50 ng/μl for 15 min). The bright field images in the right panels indicate healthy cells. High resolution enlarged images are shown in the insets. (D & E) Quantification of Annexin V staining levels (D) and Caspase 3 staining levels. (F) Western blot analysis with the indicated antibodies of cell lysates form the Ctrl-sh and sh-W3 cells after treatment with TNFα at the indicated times. The numbers below the p-AKT and the p-p38 panels indicate their respective fold change with respect to the untreated Ctrl-sh cells. All data are representative of 3 independent experiments, or are the mean (±SE; n = 3; *, p <0.05; Student's t-test).

It is well established that the resistance of many cancer cells to apoptosis and cell death is dependent on activation of the NFκB signaling pathway. Additionally, most cancer cells, including MBA-MB-231 BC cells, exhibit constitutively activated NFκB signaling and thereby can escape cell death [17]. We therefore investigated the role of WAVE3-mediated modulation of NFκB signaling in apoptosis and cell death in cancer cells. WAVE3-knockdown or non-targeting shRNA MDA-MB-231 cells were treated with the TNF*α* for 2 h, and apoptosis was assessed using flow cytometry analysis of Annexin V (apoptosis) and propidium iodide (cell death). Under these conditions, treatment of the control (Ctrl-sh) cells with TNF*α* induced little apoptosis (only 5% of the total cell population showed staining for Annexin V). In sharp contrast, the percent of apoptotic cells in WAVE3-knockdown population increased to 37% (Fig. 8A, (p<0.05)). Likewise, cell death, as measured by propidium iodide staining, was increased by more than 3.5-fold (p<0.05) in the WAVE3-knockdown population (Fig. 8B). Thus, WAVE3 is critical for resistance to apoptosis and cell death in cancer cells. This observation was further confirmed when TNF*α*-treated WAVE3-knockdown cells were assessed for apoptosis using immunocytofluorescence staining for Annexin V and Caspase 3. In the non-targeting shRNA-transfected cells (Ctrlsh), TNF*α*-treatment did not have a significant effect on apoptosis as measured by either Annexin V staining (Fig. 8C and 8D) or Caspase 3 staining (Fig. 8C and 8E). However, the WAVE3-knockdown cells were significantly sensitized (p<0.05) to apoptosis both in the untreated (Fig. 8C –TNFα panels, and Fig. 8D-E) and the TNFα-treated conditions (Fig. 8C +TNFα panels, and Fig. 8D-E).

Activation the MAP kinase pathway is one of principal components of the TNFα-mediated activation of NFκB signaling [36], and constitutive MAP kinase activation is also believed to contribute to NFκB-mediated resistance to apoptosis and cell death in cancer cells. Consistent with the effect of WAVE3-overxpression on the activation of Akt (Fig. S7 in File S1), we found that WAVE3 knockdown to inhibit the TNFα-induced Akt phosphorylation (Fig. 7F), without affecting other MAP kinases, such as p38, or JNK (Fig. S8 and Fig. S9 in File S1). Thus, our results further support the intricate WAVE3-NFκB-Akt interplay to regulate apoptosis and cell death of cancer cells.

## Discussion

Approximately 90% of the cancer deaths arise from the metastatic spread of primary tumors. Although metastases are the most clinically relevant, the process remains poorly understood at the molecular level. In our previous work, we reported a significant correlation between WAVE3 expression levels and advanced stages of BC, supporting the function of WAVE3 as a metastasis promoting protein [12], [14], [30]. We and other have also reported that WAVE3-mediated activation of cancer cell invasion and metastasis is in part through its regulation of expression and activity of key MMPs, including MMP9 [8], [10]. In this report, we sought to elucidate the WAVE3-mediated signaling events that lead to the production and activation of MMP9 in cancer cells. Previous studies have demonstrated the involvement of the NFκB pathway in the TNFα-induced MMP9 expression in several model systems [10], [37]. Accordingly, since loss of WAVE3 leads to significant downregulation of MMP9 expression and activity ([8] and Fig. 1), we speculated that similar signaling mechanisms might require WAVE3 to regulate MMP9 expression and activity. Our data demonstrate that loss of WAVE3 inhibits expression and activity of MMP9. Moreover, MMP9 expression could not be restored in the WAVE3-knockdown cells even after activation of NFκB signaling with TNFα (Fig. 1). Given the important role of NFκB signaling during cancer progression and metastasis through regulation of MMPs [38], [39], our findings identify WAVE3 as a major player in the NFκB-mediated regulation of MMP9.

In resting cells, NFκB (p65/p50 heterodimer) resides in the cytoplasm in an inactive state bound to the inhibitor IκBα. Upon activation, a signaling cascade is triggered leading to phosphorylation and degradation of IκBα, which releases p65 from its inhibitor complex, concomitant with its phosphorylation, and allows its translocation into the nucleus to activate transcription of the NFκB target genes. However, we noted that loss of WAVE3 expression affects the phosphorylation and nuclear translocation of p65, which ultimately led to the inhibition of MMP9 as well as the inhibition of invadopodia formation by cancer cells. One of the several questions that remain to be answered is which precise step(s) during the development of invadopodia is regulated by WAVE3. Since we found that WAVE3 regulated MMP9 expression-activity, invadopodia formation, and ECM degradation, WAVE3 might be involved in multiple steps during the invadopodia formation-maturation process. The net result of loss of invadopodia is the inhibition of ECM degradation which is necessary for cancer cell to invade the tumor microenvironment and initiate the invasion-metastasis cascade.

We applied a combination of genetic and pharmacological manipulations to investigate the role WAVE3 in the modulation of NFκB signaling. We showed that loss of WAVE3 results in loss of phosphorylation p65 at serine 536, which has been reported to be selectively mediated by IKKβ kinase activity [23], therefore suggesting the involvement of this kinase in the WAVE3-NFκB signaling axis. Inhibition of the proteasome (MG-132 treatment) or protein neo-synthesis (CHX treatment) had no effect on the WAVE3-mediated regulation of p65 phosphorylation levels, and therefore NFκB signaling (Fig 2), suggesting that additional protein neo-synthesis is not required for this process. Our data were also generated and were concordant in two different cell lines (MDA-MB-231 and BT549), providing support for the generality of our observations.

Our data also provide, for the first time, evidence that the WAVE3-mediated modulation of NFκB signaling, not only is specific to WAVE3; i.e., independent of the other WAVE isoforms, but also does not require the WRC, the mechanism by which WAVE3 traditionally regulates the actin-cytoskeleton. The data presented in Figure 3 clearly demonstrate that loss of WAVE3, but not that of any other member of the WRC, is responsible for the inhibition of p65 phosphorylation, excluding a major role for WRC in the regulation of the NFκB signaling. Rather, WAVE3-mediated modulation of NFκB signaling may be exerted through a much less appreciated mechanism; i.e., its adaptor/scaffolding function [40].

The role of WAVE3 in cancer cell migration and invasion is well established in the literature [7]–[9], [12], [14]–[16], [30], [41], [42]. Our published studies have clearly shown that elevated WAVE3 levels correlate positively with the invasion potential of BCs; high WAVE3 levels in more invasive cells (MDA-MB-231, BT549 and MDA-MB-435s) and low in non-invasive BC cells (MCF7, SKBr3 and T47D) [14], [15]. It has also been established that inhibition of endogenous WAVE3 levels in the aggressive cancer cell lines leads to loss of cancer invasion, both in vitro and in vivo [8], [9], [12], [14], [16] while overexpression of WAVE3 in the non-invasive cancer cells (MCF7) leads to the activation of their invasive potential [30]. The findings from the present study also establish a positive correlation between WAVE3 expression levels in BC cells and their ability to form invadopodia and degrade the ECM. Accordingly, our findings show that ability BC cells to form invadopodia and degrade ECM is inhibited by knockdown of WAVE3 in the aggressive MDA-MB-231 BC cells (Fig. 6), while this ability is enhanced by over-expression of WAVE3 in the non-invasive MCF7 BC cells (Fig. S7 in File S1).

Our study identifies another important function of WAVE3 in the NFκB pathway that is also independent from the other WAVE isoforms; i.e., loss of WAVE3 sensitizes cancer cells to apoptosis and ultimately cell death after stimulation by TNFα. In cancer cells, NFκB acts as a cell survival mechanism through the upregulation of survival genes and the inhibition pro-apoptotic genes [43]. NFκB signaling is constitutively activated in many cancers allowing them to overcome cell death and activate pro-survival pathways. Interestingly, WAVE3 expression is also increased in several cancers [6], [12], suggesting its potential contribution to the resistance of cancer cells to apoptosis. The WAVE3-mediated modulation of NFκB activity and apoptosis could also be exerted through its regulation of the LDOC1 protein, (a leucine zipper protein that is down-regulated in cancer cells). In fact, WAVE3 was reported to negatively regulate the activity of LDOC1 [44], the function of which is to inhibit NFκB activation and to sensitize cancer cells to apoptosis [45].

These novel functions of WAVE3 seem to require both the NFκB the Akt survival signaling pathways. In fact, our data show that Akt signaling is activated by the TNFα-induced stimulation of NFκB signaling (Fig. 7A). On the other hand, inhibition of the Akt signaling also has an inhibitory effect on NFκB signaling (Fig. 7A). Inhibition of either pathway results in a negative effect on invadopodia formation and ECM degradation. At this point, we are unable to determine if Akt is upstream or downstream of NFκB. The intricate interplay between these two signaling pathways and WAVE3 remains to be elucidated in future studies. Also remaining to be determined is how WAVE3 modulates the NFκB-mediated regulation of MMP9 expression and invadopodia formation. Our preliminary data using co-immunoprecipitation in combination with mass spectrometry (unpublished data) have identified several WAVE3 interacting proteins including ones involved in the regulation of the transcription machinery (FUBP2, GTF2I, MCM6 and YBOX1), and in nuclear transport (importin and exportin proteins). We therefore speculate that loss of WAVE3 may interfere with the transcription machinery and the nuclear transport systems that are required for the proper NFκB signaling.

**Figure 9:**
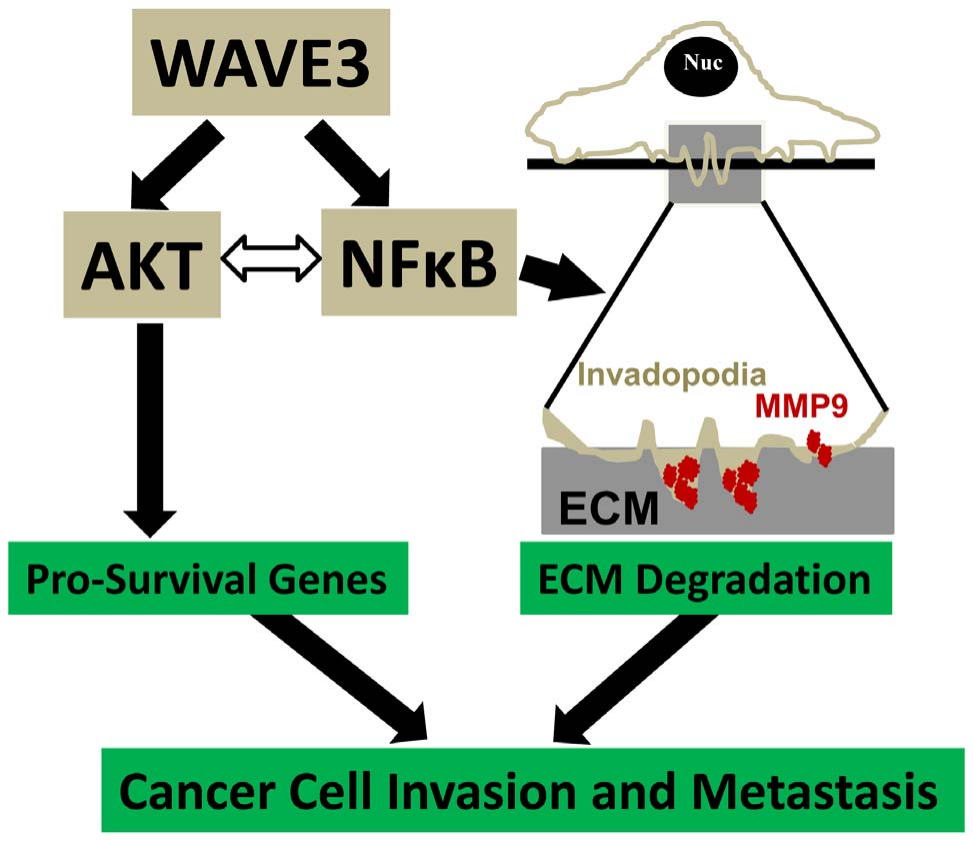
Model describing how the WAVE3-NFκB interplay modulates the molecular signaling pathways that regulate cancer invasion and metastasis. The interrelationship between WAVE3 and NFκB/Akt signaling regulates pro-survival genes to acquire chemoresistance and activates ECM degradation though the regulation of invadopodia formation and MMP9 expression and activity. The regulation of both pro-survival genes and the ECM degradation are required for the regulation of cancer cell invasion and metastasis.

In conclusion, we have identified a novel role of WAVE3 in NFκB signaling and in the invasion-metastasis cascade through its regulation of invadopodia formation and MMP9 activation. It is also interesting to note that loss of WAVE3 can sensitize cancer cells to TNFα-induced apoptosis and cell death (Fig. 9). We therefore believe that these regulatory functions of WAVE3 could be targeted to inhibit cancer cell invasion and metastasis.

## Supporting Information

File S1 **File contains Figures S1-S9.** Figure S1. NFκB promoter luciferase activity in TNFα treated *versus* untreated cells: Luciferase-based NFκB reporter assay in MDA-MB-231 (A) and BT549 (B) cells before and after treatment with TNFα. MDA-MB 231 cells were transfected with NFκB reporter, negative and positive controls, along with a Renilla luciferase reporter construct for internal normalization. After 24 h, cells were treated with 50 ng/ml of recombinant human TNFα for 15 min. Dual luciferase assays were performed in a 96-well plate and promoter activity values are expressed in arbitrary units after normalization to luciferase activity of the Renilla reporter. Experiments were performed in triplicates, and the standard deviation is indicated. All data are representative of 3 independent experiments, or are the mean (±SE; n = 3; *, p <0.05; Student's t-test). Figure S2: WAVE2 has no effect on NFκB signaling: (A) MDA-MB 231 cells were co-transfected with siWAVE2 or control siRNA and with the NFκB reporter, negative and positive controls, along with a Renilla luciferase reporter construct for internal normalization. After 24 h, cells were treated with 50 ng/ml of recombinant human TNFα for 15 min. Dual luciferase assays were performed in a 96-well plate and promoter activity values are expressed in arbitrary units after normalization to luciferase activity of the Renilla reporter. Experiments were performed in triplicates, and the standard deviation is indicated. ns, not significant. (B) Western blot analysis with the WAVE2 antibody of MDA-MB-231 cells transiently transfected with a non-targeting siRNA (Ctrl-si) or siRNA targeting either WAVE2 (W2-si). The numbers below the β-actin panel indicate the fold change of WAVE2 levels with respect to the Ctrl-si cells. β-actin was used as a loading control. All data are representative of 3 independent experiments, or are the mean (±SE; n = 3; *, p <0.05; Student's t-test). Figure S3. Knockdown of WAVE3 expression inhibits phosphorylation of p65. Western blot analysis with the indicated antibodies of cell lysates from Ctrl-sh and sh-WAVE3 BT549 cells cells treated with TNFα at the indicated times. The numbers below the β-actin panel indicate the fold change p-p65 levels with respect to the untreated Ctrl-sh cells. β-actin was used as a loading control. Figure S4. Down regulation of WAVE3 affects nuclear localization of p65: (A) Immuno-staining analysis of nuclear translocation (white arrows) of p65 protein (Red) in the Ctrl-sh (a & c) and shWAVE3 (b &d) MDA-MB-231 cells that were untreated (a & b) or treated with TNFα (c & d). Cells nuclei are counter-stained with DAPI (Blue). (B) Quantification of p65 nuclear staining. (*, p<0.05). Figure S5. Down regulation of WAVE3 inhibits MMP9 expression in BT549 cells. Western blot analysis with the indicated antibodies of cell lysates from Ctrl-sh and sh-WAVE3 BT549 cells treated with TNFα at the indicated times. The numbers below the β-actin panel indicate the fold change MMP9 levels with respect to the untreated Ctrl-sh cells. β-actin was used as a loading control. Figure S6. Colocalization of Cortactin with invadopodia in TNFα-stimulated cancer cells. Confocal microscopy micrographs of MDA-MB-231 cells grown on gelatin and treated with TNFα (50 ng/ml) for 15 min before being contained for cortactin (left panel) and F-actin filaments (middle panel). Colocalization of Cortactin with invadopodia structures is indicated by white arrow-heads. Colocalization is also clearly shown in the inset. Figure S7. Overexpression of WAVE3 in non-invasive MCF7 BC cells activates invadopodia formation and degradation of ECM stimulated by TNFα. (A) Western blot analysis with the indicated antibodies of cell lysates from MCF7 cells transiently transfected with either a pcDNA/myc-His expression vector (Empty Vector:EV) or a Myc-His-WAVE3 fusion expression vector (WAVE3), and treated with TNFα (50 ng/ml) for 15 min. β-actin was used as a loading control. (B) Confocal microscopy micrographs of control MCF7 cells (transfected with empty vector) or WAVE3-overexpressing MCF cells (WAVE3), grown on FITC-labeled gelatin. Slides were treated with TNFα (50 ng/ml) for 15 min then stained for F-actin filaments (left panels). Areas of ECM degradation are shown as black spots (middle panels). The invadopodia structures coincide with the areas of ECM degradation in the merged image (right panels). (C) Western blot analysis with the indicated antibodies of cell lysates from MCF7 cells transiently transfected with either a pcDNA/myc-His expression vector (Empty vector:EV) or a Myc-His-WAVE3 fusion expression vector (WAVE3), and treated with TNFα (50 ng/ml) for 15 min. β-actin was used as a loading control. Figure S8. (A) Loss of WAVE3 inhibits the Akt survival pathway in BT549 cells. Western blot analysis with the indicated antibodies of cell lysates from Ctrl-sh and sh-WAVE3 BT549 cells treated with TNFα at the indicated times. β-Actin was used as a loading control. Figure S9. (A) Loss of WAVE3 inhibits the Akt survival pathway in MDA-MB-231 cells. Western blot analysis with the indicated antibodies of cell lysates from Ctrl-sh and sh-WAVE3 BT549 cells treated with TNFα at the indicated times. β-Actin was used as a loading control.
(PDF)

## References

[1] Hanahan D, Weinberg RA (2011) Hallmarks of cancer: the next generation. Cell 144: 646–674 S0092-8674(11)00127-9 [pii];10.1016/j.cell.2011.02.013 [doi]. 21376230 10.1016/j.cell.2011.02.013

[2] Murphy DA, Courtneidge SA (2011) The ‘ins’ and ‘outs’ of podosomes and invadopodia: characteristics, formation and function. Nat Rev Mol Cell Biol 12: 413–426 nrm3141 [pii];10.1038/nrm3141 [doi]. 21697900 10.1038/nrm3141 PMC3423958

[3] Yamaguchi H (2012) Pathological roles of invadopodia in cancer invasion and metastasis. Eur J Cell Biol. S0171-9335(12)00081-7 [pii];10.1016/j.ejcb.2012.04.005 [doi]. 10.1016/j.ejcb.2012.04.005 22658792

[4] Kurisu S, Takenawa T (2009) The WASP and WAVE family proteins. Genome Biol 10: 226 gb-2009-10-6-226 [pii];10.1186/gb-2009-10-6-226 [doi]. 19589182 10.1186/gb-2009-10-6-226 PMC2718491

[5] Caldieri G, Ayala I, Attanasio F, Buccione R (2009) Cell and molecular biology of invadopodia. Int Rev Cell Mol Biol 275: 1–34 S1937-6448(09)75001-4 [pii];10.1016/S1937-6448(09)75001-4 [doi]. 19491051 10.1016/S1937-6448(09)75001-4

[6] Kurisu S, Takenawa T (2010) WASP and WAVE family proteins: friends or foes in cancer invasion? Cancer Sci 101: 2093–2104 CAS1654 [pii];10.1111/j.1349-7006.2010.01654.x [doi]. 20707804 10.1111/j.1349-7006.2010.01654.x PMC11158077

[7] Sossey-Alaoui K, Li X, Ranalli TA, Cowell JK (2005) WAVE3-mediated cell migration and lamellipodia formation are regulated downstream of phosphatidylinositol 3-kinase. J Biol Chem 280: 21748–21755 M500503200 [pii];10.1074/jbc.M500503200 [doi]. 15826941 10.1074/jbc.M500503200

[8] Sossey-Alaoui K, Ranalli TA, Li X, Bakin AV, Cowell JK (2005) WAVE3 promotes cell motility and invasion through the regulation of MMP-1, MMP-3, and MMP-9 expression. Exp Cell Res 308: 135–145 S0014-4827(05)00187-4 [pii];10.1016/j.yexcr.2005.04.011 [doi]. 15907837 10.1016/j.yexcr.2005.04.011

[9] Teng Y, Ren MQ, Cheney R, Sharma S, Cowell JK (2010) Inactivation of the WASF3 gene in prostate cancer cells leads to suppression of tumorigenicity and metastases. Br J Cancer 103: 1066–1075 6605850 [pii];10.1038/sj.bjc.6605850 [doi]. 20717117 10.1038/sj.bjc.6605850 PMC2965863

[10] Teng Y, Liu M, Cowell JK (2011) Functional interrelationship between the WASF3 and KISS1 metastasis-associated genes in breast cancer cells. Int J Cancer 129: 2825–2835 10.1002/ijc.25964 [doi]. 21544801 10.1002/ijc.25964 PMC3154992

[11] Fernando HS, Sanders AJ, Kynaston HG, Jiang WG (2010) WAVE3 is associated with invasiveness in prostate cancer cells. Urol Oncol 28: 320–327 S1078-1439(08)00381-5 [pii];10.1016/j.urolonc.2008.12.022 [doi]. 19395286 10.1016/j.urolonc.2008.12.022

[12] Sossey-Alaoui K, Safina A, Li X, Vaughan MM, Hicks DG, et al (2007) Down-regulation of WAVE3, a metastasis promoter gene, inhibits invasion and metastasis of breast cancer cells. Am J Pathol 170: 2112–2121 S0002-9440(10)61418-6 [pii];10.2353/ajpath.2007.060975 [doi]. 17525277 10.2353/ajpath.2007.060975 PMC1899429

[13] Zhang Y, Guan XY, Dong B, Zhao M, Wu JH, et al.. (2012) Expression of MMP-9 and WAVE3 in colorectal cancer and its relationship to clinicopathological features. J Cancer Res Clin Oncol. 10.1007/s00432-012-1274-3 [doi]. 10.1007/s00432-012-1274-3 PMC11824433 22806308

[14] Kulkarni S, Augoff K, Rivera L, McCue B, Khoury T, et al (2012) Increased Expression Levels of WAVE3 Are Associated with the Progression and Metastasis of Triple Negative Breast Cancer. PLOS ONE 7: e42895 10.1371/journal.pone.0042895 [doi];PONE-D-12-13453 [pii]. 22952619 10.1371/journal.pone.0042895 PMC3428347

[15] Sossey-Alaoui K (2013) Surfing the big WAVE: Insights into the role of WAVE3 as a driving force in cancer progression and metastasis. Semin Cell Dev Biol 24: 287–297 S1084-9521(12)00186-3 [pii];10.1016/j.semcdb.2012.10.006 [doi]. 23116924 10.1016/j.semcdb.2012.10.006 PMC4207066

[16] Taylor MA, Davuluri G, Parvani JG, Schiemann BJ, Wendt MK, et al (2013) Upregulated WAVE3 expression is essential for TGF-beta-mediated EMT and metastasis of triple-negative breast cancer cells. Breast Cancer Res Treat 142: 341–353 10.1007/s10549-013-2753-1 [doi]. 24197660 10.1007/s10549-013-2753-1 PMC3888319

[17] Chaturvedi MM, Sung B, Yadav VR, Kannappan R, Aggarwal BB (2011) NF-kappaB addiction and its role in cancer: ‘one size does not fit all’. Oncogene 30: 1615–1630 onc2010566 [pii];10.1038/onc.2010.566 [doi]. 21170083 10.1038/onc.2010.566 PMC3141287

[18] Wu Y, Zhou BP (2010) TNF-alpha/NF-kappaB/Snail pathway in cancer cell migration and invasion. Br J Cancer 102: 639–644 6605530 [pii];10.1038/sj.bjc.6605530 [doi]. 20087353 10.1038/sj.bjc.6605530 PMC2837572

[19] Ghosh S, Karin M (2002) Missing pieces in the NF-kappaB puzzle. Cell 109 Suppl: S81–S96. S0092867402007031 [pii]. 10.1016/s0092-8674(02)00703-1 11983155

[20] O'Dea E, Hoffmann A (2009) NF-kappaB signaling. Wiley Interdiscip Rev Syst Biol Med 1: 107–115 10.1002/wsbm.30 [doi]. 20151024 10.1002/wsbm.30 PMC2819170

[21] Balkwill F (2009) Tumour necrosis factor and cancer. Nat Rev Cancer 9: 361–371 nrc2628 [pii];10.1038/nrc2628 [doi]. 19343034 10.1038/nrc2628

[22] O'Dea E, Hoffmann A (2010) The regulatory logic of the NF-kappaB signaling system. Cold Spring Harb Perspect Biol 2: a000216 10.1101/cshperspect.a000216 [doi]. 20182598 10.1101/cshperspect.a000216 PMC2827908

[23] Mattioli I, Sebald A, Bucher C, Charles RP, Nakano H, et al (2004) Transient and selective NF-kappa B p65 serine 536 phosphorylation induced by T cell costimulation is mediated by I kappa B kinase beta and controls the kinetics of p65 nuclear import. J Immunol 172: 6336–6344. 15128824 10.4049/jimmunol.172.10.6336

[24] Oner C, Schatz F, Kizilay G, Murk W, Buchwalder LF, et al (2008) Progestin-inflammatory cytokine interactions affect matrix metalloproteinase-1 and -3 expression in term decidual cells: implications for treatment of chorioamnionitis-induced preterm delivery. J Clin Endocrinol Metab 93: 252–259 jc.2007-1538 [pii];10.1210/jc.2007-1538 [doi]. 17940116 10.1210/jc.2007-1538 PMC2190749

[25] Sato H, Kita M, Seiki M (1993) v-Src activates the expression of 92-kDa type IV collagenase gene through the AP-1 site and the GT box homologous to retinoblastoma control elements. A mechanism regulating gene expression independent of that by inflammatory cytokines. J Biol Chem 268: 23460–23468. 8226872

[26] Fortunato SJ, Menon R, Lombardi SJ (2002) Role of tumor necrosis factor-alpha in the premature rupture of membranes and preterm labor pathways. Am J Obstet Gynecol 187: 1159–1162. S0002937802003848 [pii]. 10.1067/mob.2002.127457 12439495

[27] Bialkowska K, Ma YQ, Bledzka K, Sossey-Alaoui K, Izem L, et al (2010) The integrin co-activator Kindlin-3 is expressed and functional in a non-hematopoietic cell, the endothelial cell. J Biol Chem 285: 18640–18649 M109.085746 [pii];10.1074/jbc.M109.085746 [doi]. 20378539 10.1074/jbc.M109.085746 PMC2881789

[28] Sossey-Alaoui K, Pluskota E, Davuluri G, Bialkowska K, Das M, et al (2014) Kindlin-3 enhances breast cancer progression and metastasis by activating Twist-mediated angiogenesis. FASEB J 28: 2260–2271 fj.13-244004 [pii];10.1096/fj.13-244004 [doi]. 24469992 10.1096/fj.13-244004 PMC3986835

[29] Stovold CF, Millard TH, Machesky LM (2005) Inclusion of Scar/WAVE3 in a similar complex to Scar/WAVE1 and 2. BMC Cell Biol 6: 11 1471-2121-6-11 [pii];10.1186/1471-2121-6-11 [doi]. 15752430 10.1186/1471-2121-6-11 PMC555569

[30] Sossey-Alaoui K, Downs-Kelly E, Das M, Izem L, Tubbs R, et al (2011) WAVE3, an actin remodeling protein, is regulated by the metastasis suppressor microRNA, miR-31, during the invasion-metastasis cascade. Int J Cancer 129: 1331–1343 10.1002/ijc.25793 [doi]. 21105030 10.1002/ijc.25793 PMC3081370

[31] Yamaguchi H, Yoshida S, Muroi E, Yoshida N, Kawamura M, et al (2011) Phosphoinositide 3-kinase signaling pathway mediated by p110alpha regulates invadopodia formation. J Cell Biol 193: 1275–1288 jcb.201009126 [pii];10.1083/jcb.201009126 [doi]. 21708979 10.1083/jcb.201009126 PMC3216328

[32] Agarwal E, Chaudhuri A, Leiphrakpam PD, Haferbier KL, Brattain MG, et al (2014) Akt inhibitor MK-2206 promotes anti-tumor activity and cell death by modulation of AIF and Ezrin in colorectal cancer. BMC Cancer 14: 145 1471-2407-14-145 [pii];10.1186/1471-2407-14-145 [doi]. 24581231 10.1186/1471-2407-14-145 PMC3941258

[33] Cheng Y, Zhang Y, Zhang L, Ren X, Huber-Keener KJ, et al (2012) MK-2206, a novel allosteric inhibitor of Akt, synergizes with gefitinib against malignant glioma via modulating both autophagy and apoptosis. Mol Cancer Ther 11: 154–164 1535-7163.MCT-11-0606 [pii];10.1158/1535-7163.MCT-11-0606 [doi]. 22057914 10.1158/1535-7163.MCT-11-0606 PMC3302182

[34] Lai YC, Liu Y, Jacobs R, Rider MH (2012) A novel PKB/Akt inhibitor, MK-2206, effectively inhibits insulin-stimulated glucose metabolism and protein synthesis in isolated rat skeletal muscle. Biochem J 447: 137–147 BJ20120772 [pii];10.1042/BJ20120772 [doi]. 22793019 10.1042/BJ20120772

[35] Liu R, Liu D, Trink E, Bojdani E, Ning G, et al (2011) The Akt-specific inhibitor MK2206 selectively inhibits thyroid cancer cells harboring mutations that can activate the PI3K/Akt pathway. J Clin Endocrinol Metab 96: E577–E585 jc.2010-2644 [pii];10.1210/jc.2010-2644 [doi]. 21289267 10.1210/jc.2010-2644 PMC3070256

[36] Kant S, Swat W, Zhang S, Zhang ZY, Neel BG, et al (2011) TNF-stimulated MAP kinase activation mediated by a Rho family GTPase signaling pathway. Genes Dev 25: 2069–2078 25/19/2069 [pii];10.1101/gad.17224711 [doi]. 21979919 10.1101/gad.17224711 PMC3197205

[37] Cho SG, Li D, Stafford LJ, Luo J, Rodriguez-Villanueva M, et al (2009) KiSS1 suppresses TNFalpha-induced breast cancer cell invasion via an inhibition of RhoA-mediated NF-kappaB activation. J Cell Biochem 107: 1139–1149 10.1002/jcb.22216 [doi]. 19533666 10.1002/jcb.22216 PMC2745330

[38] Deryugina EI, Quigley JP (2006) Matrix metalloproteinases and tumor metastasis. Cancer Metastasis Rev 25: 9–34 10.1007/s10555-006-7886-9 [doi]. 16680569 10.1007/s10555-006-7886-9

[39] Naugler WE, Karin M (2008) NF-kappaB and cancer-identifying targets and mechanisms. Curr Opin Genet Dev 18: 19–26 S0959-437X(08)00025-7 [pii];10.1016/j.gde.2008.01.020 [doi]. 18440219 10.1016/j.gde.2008.01.020 PMC2587362

[40] Scott JD (2003) A-kinase-anchoring proteins and cytoskeletal signalling events. Biochem Soc Trans 31: 87–89 10.1042/[doi]. 12546660 10.1042/bst0310087

[41] Sossey-Alaoui K, Li X, Cowell JK (2007) c-Abl-mediated phosphorylation of WAVE3 is required for lamellipodia formation and cell migration. J Biol Chem 282: 26257–26265 M701484200 [pii];10.1074/jbc.M701484200 [doi]. 17623672 10.1074/jbc.M701484200

[42] Sossey-Alaoui K, Bialkowska K, Plow EF (2009) The miR200 family of microRNAs regulates WAVE3-dependent cancer cell invasion. J Biol Chem 284: 33019–33029 M109.034553 [pii];10.1074/jbc.M109.034553 [doi]. 19801681 10.1074/jbc.M109.034553 PMC2785142

[43] Deorukhkar A, Krishnan S (2010) Targeting inflammatory pathways for tumor radiosensitization. Biochem Pharmacol 80: 1904–1914 S0006-2952(10)00473-9 [pii];10.1016/j.bcp.2010.06.039 [doi]. 20599771 10.1016/j.bcp.2010.06.039 PMC3090731

[44] Mizutani K, Koike D, Suetsugu S, Takenawa T (2005) WAVE3 functions as a negative regulator of LDOC1. J Biochem 138: 639–646 138/5/639 [pii];10.1093/jb/mvi160 [doi]. 16272576 10.1093/jb/mvi160

[45] Nagasaki K, Schem C, von KC, Biallek M, Rosel F, et al (2003) Leucine-zipper protein, LDOC1, inhibits NF-kappaB activation and sensitizes pancreatic cancer cells to apoptosis. Int J Cancer 105: 454–458 10.1002/ijc.11122 [doi]. 12712434 10.1002/ijc.11122

