# Supplementary material for: WAVE3-NFκB Interplay Is Essential for the Survival and Invasion of Cancer Cells
Source: PLoS One. 2014 Oct 16;9(10):e110627. doi: 10.1371/journal.pone.0110627 (PMC4199728; doi:10.1371/journal.pone.0110627)
Supplement: File S1 — File contains Figures S1-S9. Figure S1. NFκB promoter luciferase activity in TNFα treated versus untreated cells: Luciferase-based NFκB reporter assay in MDA-MB-231 (A) and BT549 (B) cells before and after treatment with TNFα. MDA-MB 231 cells were transfected with NFκB reporter, negative and positive controls, along with a Renilla luciferase reporter construct for internal normalization. After 24 h, cells were treated with 50 ng/ml of recombinant human TNFα for 15 min. Dual luciferase assays were performed in a 96-well plate and promoter activity values are expressed in arbitrary units after normalization to luciferase activity of the Renilla reporter. Experiments were performed in triplicates, and the standard deviation is indicated. All data are representative of 3 independent experiments, or are the mean (±SE; n = 3; *, p <0.05; Student's t-test). Figure S2: WAVE2 has no effect on NFκB signaling: (A) MDA-MB 231 cells were co-transfected with siWAVE2 or control siRNA and with the NFκB reporter, negative and positive controls, along with a Renilla luciferase reporter construct for internal normalization. After 24 h, cells were treated with 50 ng/ml of recombinant human TNFα for 15 min. Dual luciferase assays were performed in a 96-well plate and promoter activity values are expressed in arbitrary units after normalization to luciferase activity of the Renilla reporter. Experiments were performed in triplicates, and the standard deviation is indicated. ns, not significant. (B) Western blot analysis with the WAVE2 antibody of MDA-MB-231 cells transiently transfected with a non-targeting siRNA (Ctrl-si) or siRNA targeting either WAVE2 (W2-si). The numbers below the β-actin panel indicate the fold change of WAVE2 levels with respect to the Ctrl-si cells. β-actin was used as a loading control. All data are representative of 3 independent experiments, or are the mean (±SE; n = 3; *, p <0.05; Student's t-test). Figure S3. Knockdown of WAVE3 expression inhibits phosphoryl [file pone.0110627.s001.pdf]

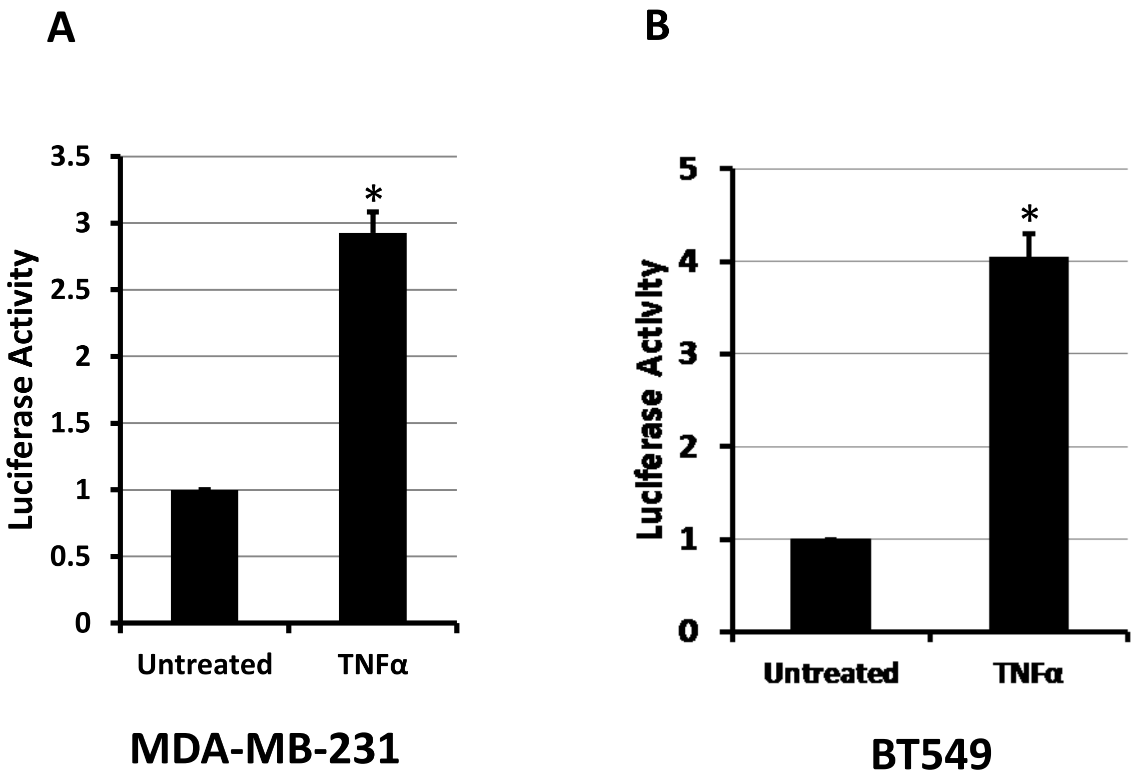
A
3.5
3
2.5
2
1.5
1
0.5
0
Luciferase Activity
*
Untreated
TNFα
MDA-MB-231
B
5
4
3
2
1
0
Luciferase Activity
*
Untreated
TNFα
BT549

Figure S1

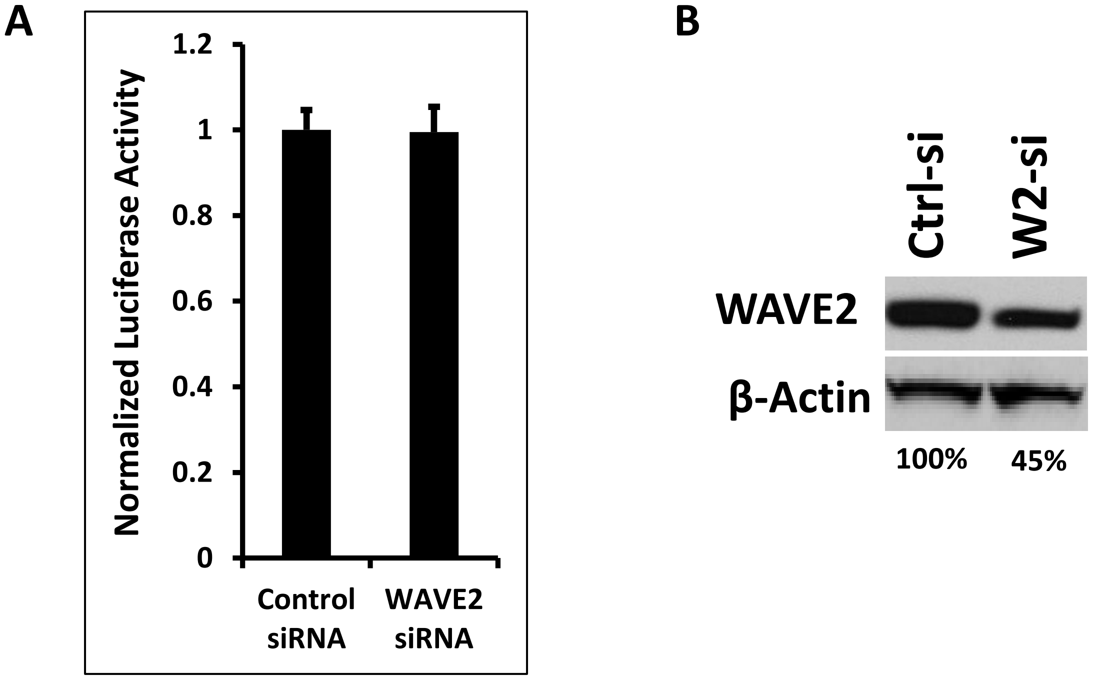
A
1.2
1
0.8
0.6
0.4
0.2
0
Normalized Luciferase Activity
Control siRNA
WAVE2 siRNA
B
Ctrl-si
W2-si
WAVE2
β-Actin
100%
45%

Figure S2

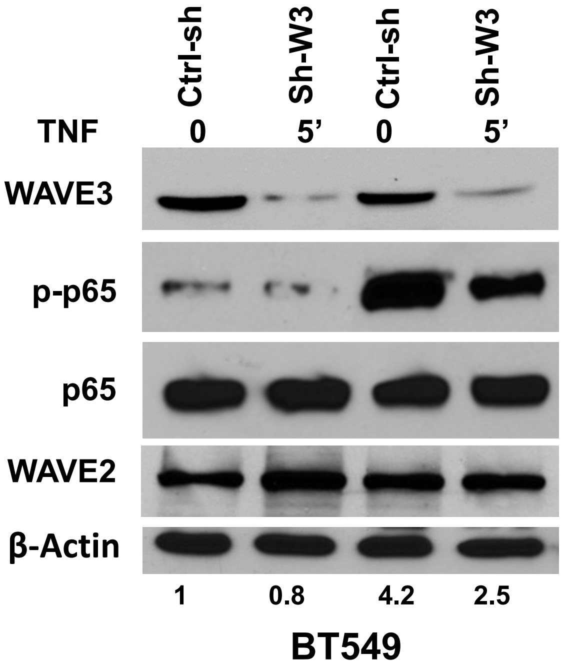
Ctrl-sh
Sh-W3
Ctrl-sh
Sh-W3
TNF
0
5'
0
5'
WAVE3
p-p65
p65
WAVE2
β-Actin
1
0.8
4.2
2.5
BT549

Figure 3

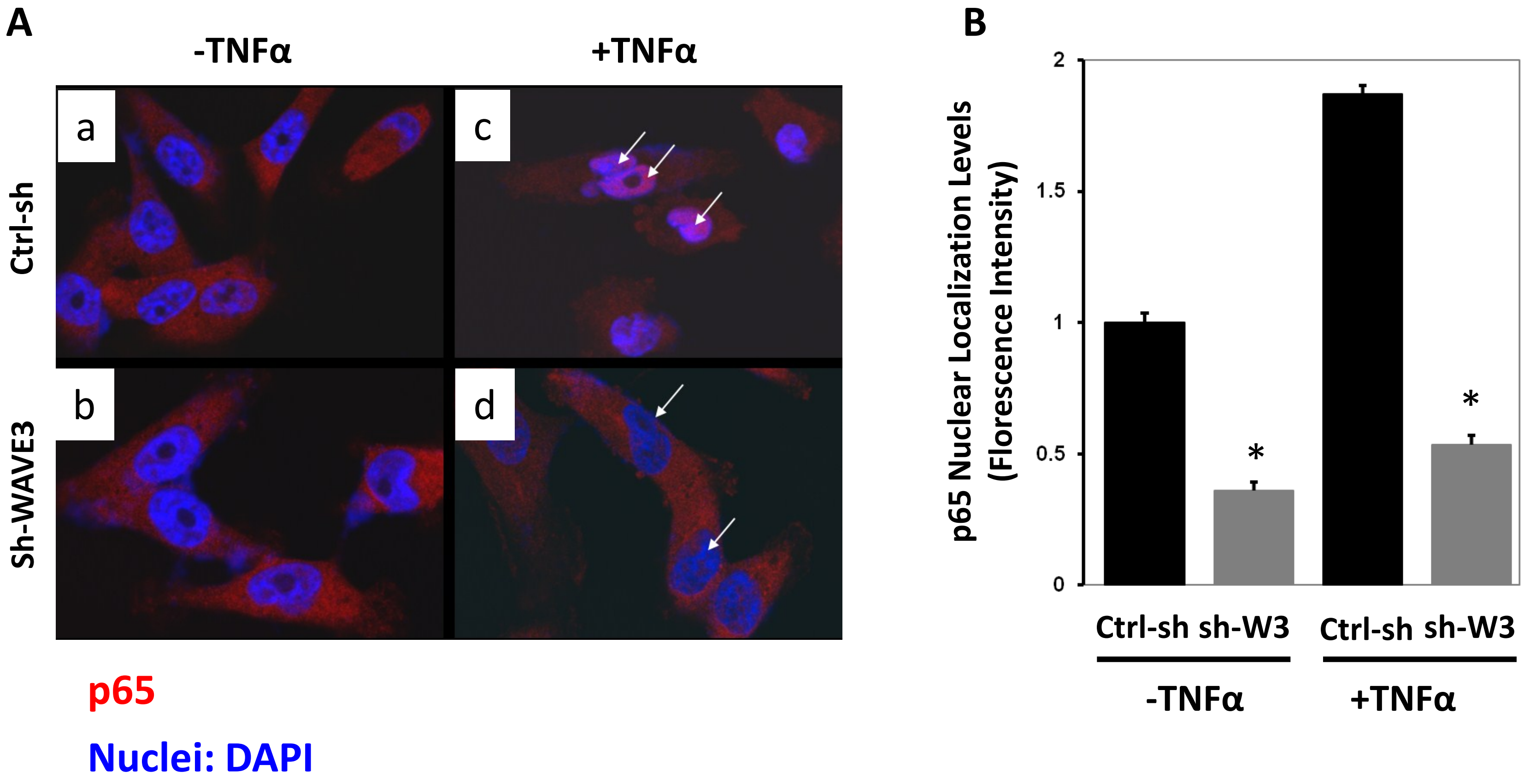
A
-TNFα
+TNFα
Ctrl-sh
Sh-WAVE3
a
b
c
d
p65
Nuclei: DAPI
B
p65 Nuclear Localization Levels (Florescence Intensity)
0
0.5
1
1.5
2
*
*
Ctrl-sh sh-W3
Ctrl-sh sh-W3
-TNFα
+TNFα

Figure S4

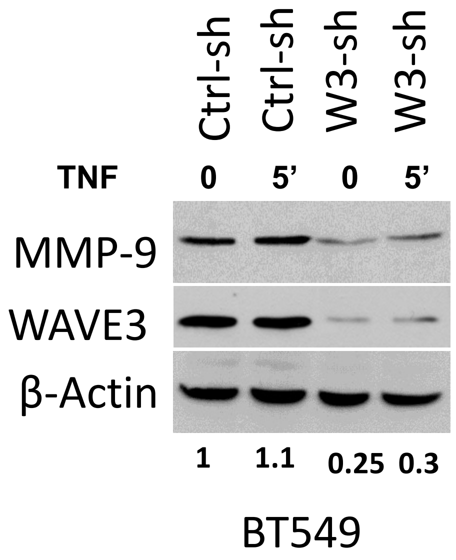
Ctrl-sh
Ctrl-sh
W3-sh
W3-sh
TNF
0
5’
0
5’
MMP-9
WAVE3
β-Actin
1
1.1
0.25
0.3
BT549

Figure S5

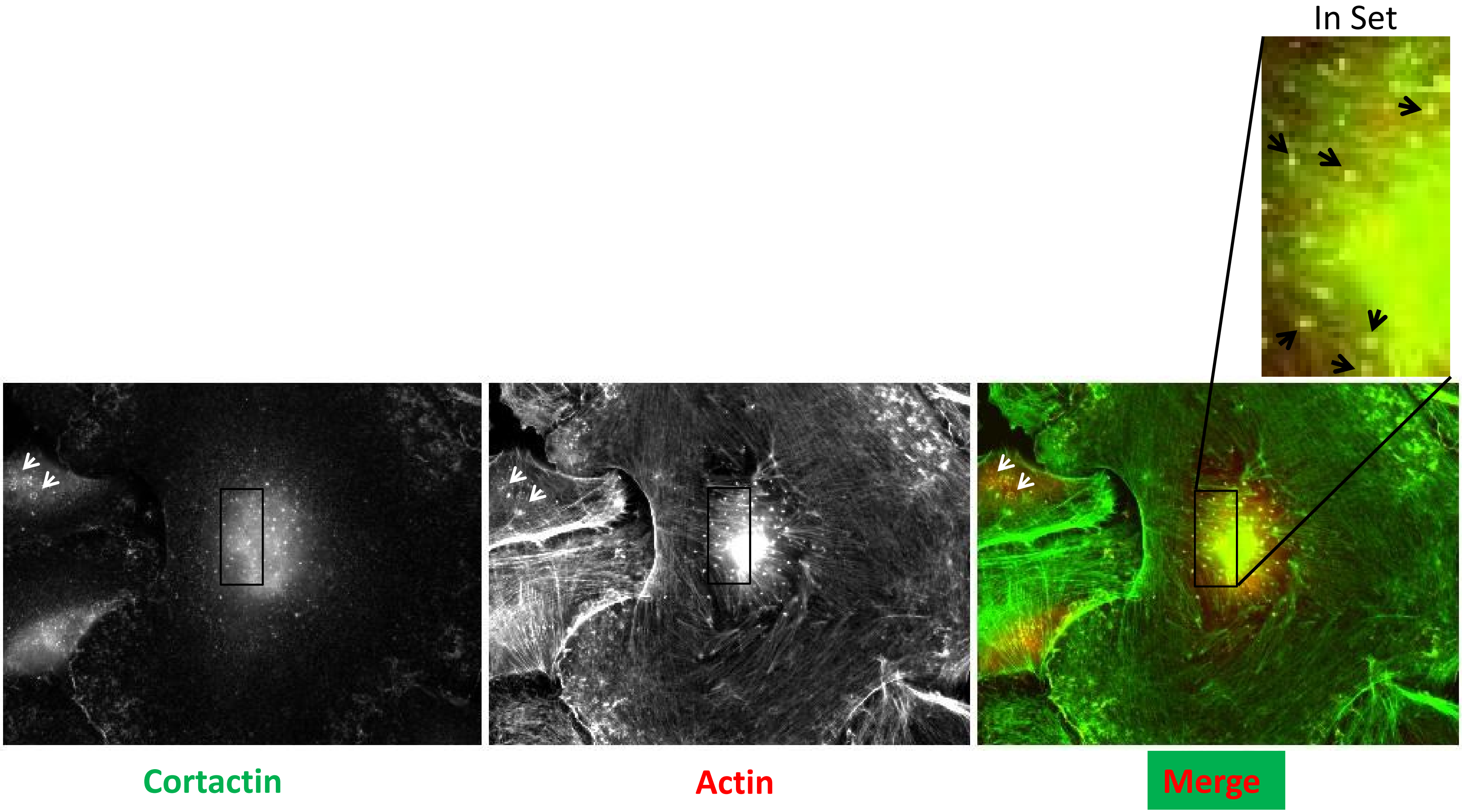

Figure S6

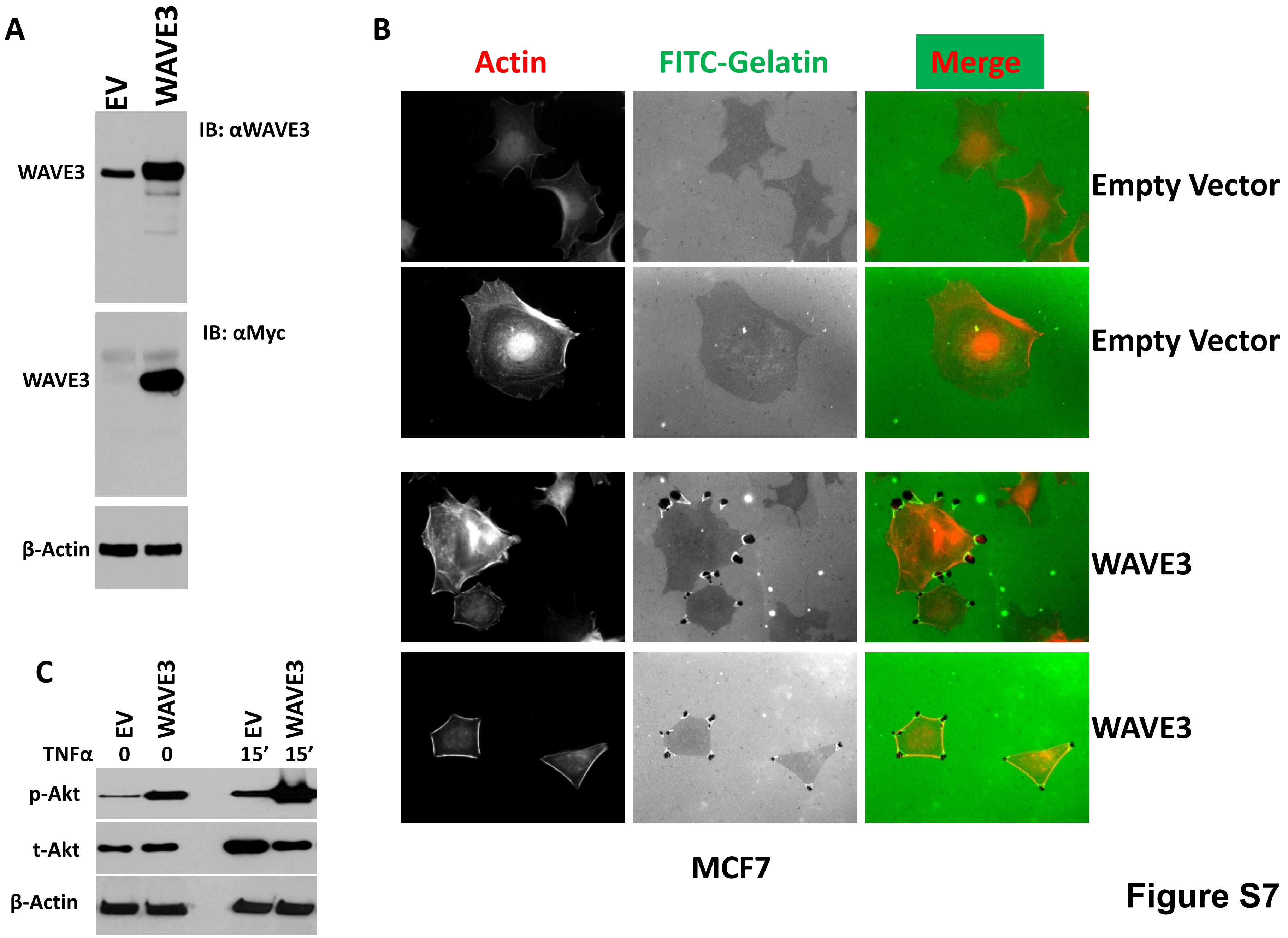
A
EV
WAVE3
IB: αWAVE3
WAVE3
IB: αMyc
WAVE3
β-Actin
B
Actin
FITC-Gelatin
Merge
Empty Vector
Empty Vector
WAVE3
WAVE3
MCF7
C
EV
WAVE3
EV
WAVE3
TNFα
0
0
15’
15’
p-Akt
t-Akt
β-Actin

Figure S7

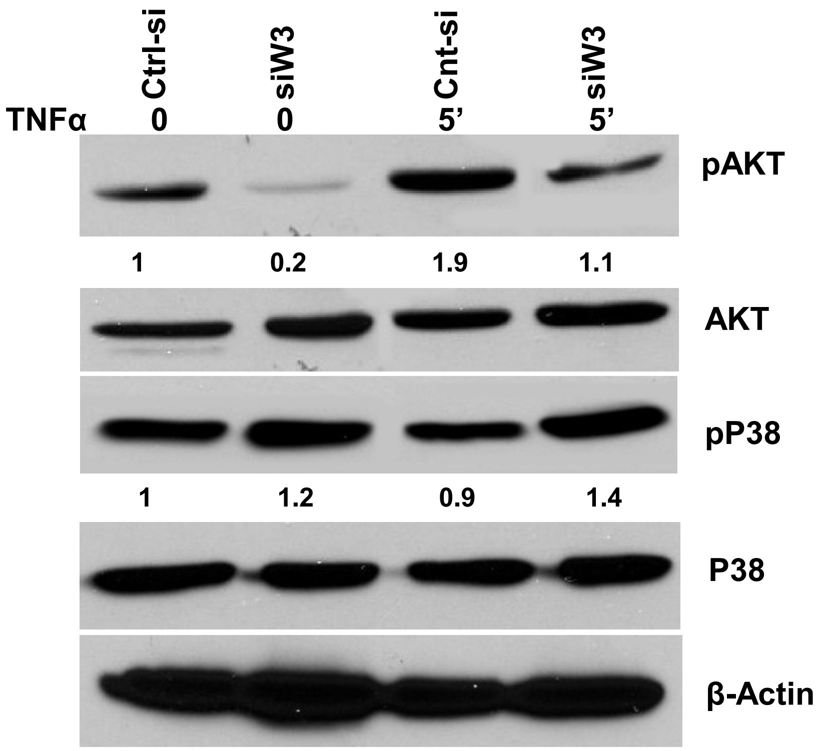

BT549 cells

Figure S8

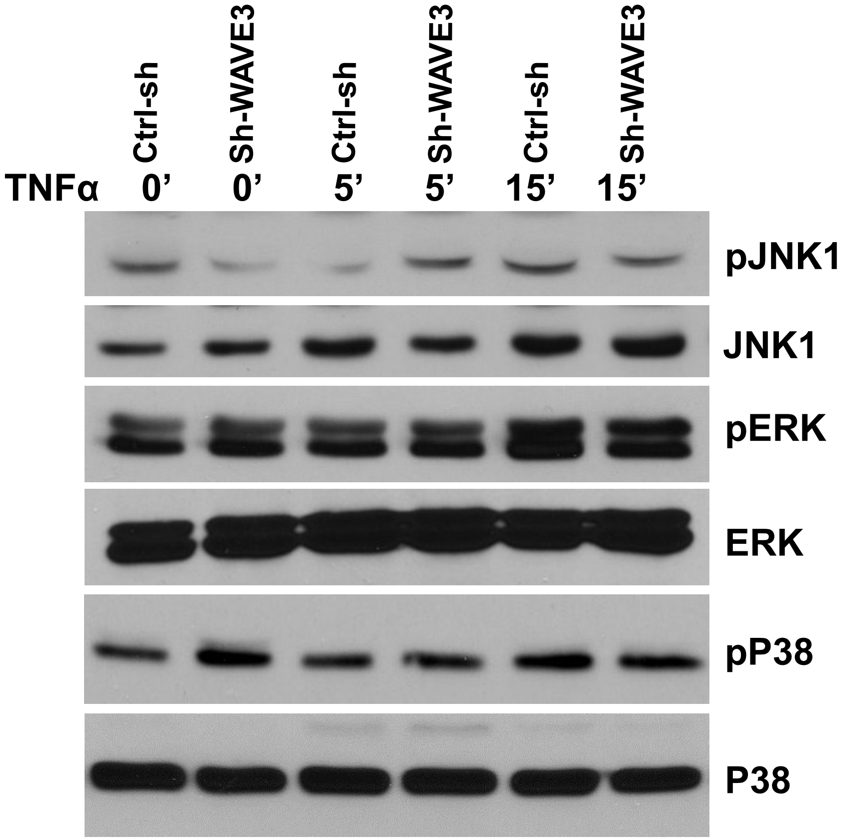

MDA-MB-231

Figure S9

SUPPLEMENTAL FIGURES LEGENDS

Figure S1: NFκB promoter luciferase activity in TNFα treated *versus* untreated cells. Luciferase-based NFκB reporter assay in MDA-MB-231 (A) and BT549 (B) cells before and after treatment with TNFα. MDA-MB 231 cells were transfected with NFκB reporter, negative and positive controls, along with a Renilla luciferase reporter construct for internal normalization. After 24 h, cells were treated with 50 ng/ml of recombinant human TNFα for 15 min. Dual luciferase assays were performed in a 96-well plate and promoter activity values are expressed in arbitrary units after normalization to luciferase activity of the Renilla reporter. Experiments were performed in triplicates, and the standard deviations are indicated. All data are representative of 3 independent experiments, or are the mean (±SD; n=3; *, $p < 0.05$; Student's t-test).

Figure S2: WAVE2 has no effect on NFκB signaling.

(A) MDA-MB 231 cells were co-transfected with siWAVE2 or control siRNA and with the NFκB reporter, negative and positive controls, along with a Renilla luciferase reporter construct for internal normalization. After 24 h, cells were treated with 50 ng/ml of recombinant human TNFα for 15 min. Dual luciferase assays were performed in a 96-well plate and promoter activity values are expressed in arbitrary units after normalization to luciferase activity of the Renilla reporter. Experiments were performed in triplicates, and the standard deviations are indicated (ns, not significant). (B) Western blot analysis with WAVE2 antibody of MDA-MB-231 cells transiently transfected with a non-targeting siRNA (Ctrl-si) or siRNA targeting WAVE2 (W2-si). β-actin was used as a loading control. The numbers below the β-actin panel indicate the fold change of WAVE2 levels with respect to the Ctrl-si cells. All data are representative of 3 independent experiments and are the means ±SD (n=3; *, $p < 0.05$; Student's t-test).

Figure S3. Knockdown of WAVE3 expression inhibits phosphorylation of p65. Western blot analysis with the indicated antibodies of cell lysates from Ctrl-sh and sh-WAVE3 BT549 cells cells treated with TNFα at the indicated times. β-actin was used as a loading control. The numbers below the β-actin panel indicate the fold change p-p65 levels with respect to the untreated Ctrl-sh cells.

Figure S4. Down-regulation of WAVE3 affects nuclear localization of p65. (A) Immuno-staining analysis of nuclear translocation (white arrows) of p65 protein (Red) in the Ctrl-sh (a & c) and shWAVE3 (b &d) MDA-MB-231 cells that were untreated (a & b) or treated with TNFα (c & d). Cells nuclei are counter-stained with DAPI (Blue). (B) Quantification of p65 nuclear staining. (*, $p<0.05$).

Figure S5. Down-regulation of WAVE3 inhibits MMP9 expression in BT549 cells. Western blot analysis with the indicated antibodies of cell lysates from Ctrl-sh and sh-WAVE3 BT549 cells treated with TNFα at the indicated times. β-actin was used as a loading control.The numbers below the β-actin panel indicate the fold change MMP9 levels with respect to the untreated Ctrl-sh cells.

Figure S6. Colocalization of Cortactin with invadopodia in TNFα-stimulated cancer cells.
Confocal microscopic micrographs of MDA-MB-231 cells grown on gelatin and treated with TNFα (50 ng/ml) for 15 min before being stained for Cortactin (left panel) and F-actin filaments (middle panel). Colocalization of Cortactin with invadopodia structures is indicated by white arrow-heads. Colocalization is also clearly shown in the inset.

Figure S7. Overexpression of WAVE3 in non-invasive MCF7 BC cells activates invadopodia formation and degradation of ECM stimulated by TNFα.
(A) Western blot analysis with the indicated antibodies of cell lysates from MCF7 cells transiently transfected with either a pcDNA/myc-His expression vector (Empty Vector:EV) or a Myc-His-WAVE3 fusion expression vector (WAVE3), and treated with TNFα (50 ng/ml) for 15 min. β-actin was used as a loading control. (B) Confocal microscopic micrographs of control MCF7 cells (transfected with empty vector) or WAVE3-overexpressing MCF cells (WAVE3),

grown on FITC-labeled gelatin. Slides were treated with TNFα (50 ng/ml) for 15 min then stained for F-actin filaments (left panels). Areas of ECM degradation are shown as black spots (middle panels). The invadopodia structures coincide with the areas of ECM degradation in the merged image (right panels). (C) Western blot analysis with the indicated antibodies of cell lysates from MCF7 cells transiently transfected with either a pcDNA/myc-His expression vector (Empty vector:EV) or a Myc-His-WAVE3 fusion expression vector (WAVE3), and treated with TNFα (50 ng/ml) for 15 min. β-actin was used as a loading control.

Figure S8. Loss of WAVE3 inhibits the Akt survival pathway in BT549 cells. Western blot analysis with the indicated antibodies of cell lysates from Ctrl-sh and sh-WAVE3 BT549 cells treated with TNFα at the indicated times. β-Actin was used as a loading control.

Figure S9. Loss of WAVE3 inhibits the Akt survival pathway in MDA-MB-231 cells. (A)Western blot analysis with the indicated antibodies of cell lysates from Ctrl-sh and sh-WAVE3 BT549 cells treated with TNFα at the indicated times. β-Actin was used as a loading control.
